# Heat stress tolerance in peas (*Pisum sativum* L.): Current status and way forward

**DOI:** 10.3389/fpls.2022.1108276

**Published:** 2023-01-17

**Authors:** Jyoti Devi, Vidya Sagar, Gyan P. Mishra, Prakash Kumar Jha, Nakul Gupta, Rakesh K. Dubey, Prabhakar M. Singh, Tusar K. Behera, P. V. Vara Prasad

**Affiliations:** ^1^ Indian Council of Agricultural Research-Indian Institute of Vegetable Research, Jakhini, Varanasi, India; ^2^ Indian Council of Agricultural Research-Indian Agricultural Research Institute, Pusa, New Delhi, India; ^3^ Feed the Future Innovation Lab for Collaborative Research on Sustainable Intensification, Kansas State University, Manhattan, KS, United States; ^4^ Department of Agronomy, Kansas State University, Manhattan, KS, United States

**Keywords:** heat stress, *Pisum*, heat shock proteins (HSPs), threshold temperature, QTLs, breeding

## Abstract

In the era of climate change, the overall productivity of pea (*Pisum sativum* L.) is being threatened by several abiotic stresses including heat stress (HS). HS causes severe yield losses by adversely affecting several traits in peas. A reduction in pod yield has been reported from 11.1% to 17.5% when mean daily temperature increase from 1.4 to 2.2°C. High-temperature stress (30.5-33°C) especially during reproductive phase is known to drastically reduce both seed yield and germination. HS during germination and early vegetative stage resulted in poor emergence and stunted plant growth along with detrimental effects on physiological functions of the pea plant. To combat HS and continue its life cycle, plants use various defense strategies including heat escape, avoidance or tolerance mechanisms. Ironically, the threshold temperatures for pea plant and its responses are inconsistent and not yet clearly identified. Trait discovery through traditional breeding such as semi leaflessness (*afila*), upright growing habit, lodging tolerance, lower canopy temperature and small seeded nature has highlighted their utility for greater adaptation under HS in pea. Screening of crop gene pool and landraces for HS tolerance in a targeted environment is a simple approach to identify HS tolerant genotypes. Thus, precise phenotyping using modern phenomics tools could lead to increased breeding efficiency. The NGS (next generation sequencing) data can be associated to find the candidate genes responsible for the HS tolerance in pea. In addition, genomic selection, genome wide association studies (GWAS) and marker assisted selection (MAS) can be used for the development of HS tolerant pea genotypes. Additionally, development of transgenics could be an alternative strategy for the development of HS tolerant pea genotypes. This review comprehensively covers the various aspects of HS tolerance mechanisms in the pea plant, screening protocols, omic advances, and future challenges for the development of HS tolerant genotypes.

## 1 Introduction

With its domestication history of nearly 10,000 years ago, pea (*Pisum sativum* L.) is one of the leading annual legumes of the world, cultivated over an area of 7.18 and 2.78 m ha for dry and green seeds, respectively (FAOSTAT, 2019). It was one of the first genetic model legumes used to learn about basic genetic principles in 1865 (Mendel, 1865). Peas have balance of micro and macro nutrition profile along with high dietary fiber, antioxidants, and numerous important biomolecules, thus have health benefits in managing diabetes, cardio problems, certain cancers, and many degenerative diseases (Kumari and Deka, 2021). Historically, it is a cool season crop, but its area is now extending to warmers regions of the world due to the development of cultivars more resilient to certain abiotic stresses (Bueckert et al., 2015). It is the fourth important cultivated legume (9.96 m ha) globally, after common beans (*Phaseolus vulgaris* L.; 33 m ha), cowpeas (*Vigna unguiculata* L.; 14.4 m ha) and chickpeas (*Cicer arietinum* L.; 13.7 m ha) (FAOSTAT, 2019). Despite a substantial increase in area (from 6.9 to 7.2 m ha of dry peas; 1.6 to 2.8 m ha of green peas) and production (from 10.4 to 14.2 m t of dry peas; 12.4 to 21.7 m t of green peas), a slight shift has been recorded in pea productivity (from 1.5 to 2.0 t for dry peas; 7.7 to 7.8 t/ha for green peas) during last two decade *viz*., from 2001 to 2019 (FAOSTAT, 2019). Increasing the crop productivity to meet the world’s burgeoning populations food needs, in the presence of various biotic and abiotic stresses has become the major challenge for the crop scientists and producers.

Climate change has shifted the interest of the pea breeders to breed climate resilient high yielding cultivars suitable for varying climatic conditions. Furthermore, crop sensitivity to climate change is broadly contributed by crop responses to temperature, precipitation and rise in atmospheric carbon dioxide (CO_2_) and its impact on crop productivity (Kaushal et al., 2016; Zhao et al., 2017). Heat stress (HS) has the negative impact on the yield as it is the key environmental factor that regulates the growth and developmental processes. Each plant species has its own maximum, optimum and minimum temperature range for growth and development, known as cardinal temperatures (Wahid et al., 2007) and HS occurs when there is a rise in the soil and air temperature beyond optimum threshold(s) for certain time which causes damage to physiology, growth, development, and yield. HS response has been defined as a complex function of intensity (temperature in degrees), duration of exposure, rate of increase and timing of stress. In general, a transient elevation in temperature, usually 10-15°C above ambient, is considered heat shock or HS to the plants (Wahid et al., 2007). Being adapted to cooler climate, pea requires mean seasonal temperature of 10-18°C for its optimum growth. In addition, peas have a lower HS tolerance than other winter legumes such as chickpea and lentil (*Lens culinaris* L.) (Kumar et al., 2021), and its productivity usually declines when the maximum day temperature during flowering exceeds 25°C (Guilioni et al., 1997; Sadras et al., 2013). Moreover, optimum temperature at critical growth stages for pea is key for the realization of higher yield. Contrary to this, adverse temperature could result in deleterious effects on physiological processes including photosynthesis, respiration, reproduction, biomass accumulation and ultimately reduction in the grain yield. Ridge and Pye (1985) reported that each 1°C rise in mean temperature during flowering, may reduce the production by 0.6 t/ha in a number of pea genotypes. In India, a reduction of 0.7 to 0.8 t/ha has been reported (Lamichaney et al., 2021). Hence, there is a need to develop more climate resilient pea genotypes which can perform better under HS conditions.

Many reviews covering various legumes have highlighted the impact of HS including the strategies to breed HS tolerant genotypes as most relevant approach for adaptation to stress (Sita et al., 2017; Liu et al., 2019; Janni et al., 2020; Kumar et al., 2020; Kumar et al., 2021). Although, a few independent studies in pea (Sadras et al., 2013; Jiang et al., 2015; Jiang et al., 2017; Tafesse, 2018; Jiang et al., 2020; Mohapatra et al., 2020; Tafesse et al., 2020; Tafesse et al., 2021; Lamichaney et al., 2021) have demonstrated the negative effects of increased temperature on yield; but they are not comprehensively summarized. Identification of traits controlling any adaptive response of cultivars to HS is an essential step for effective breeding and selection of HS tolerant pea cultivars. This could lead to flexibility in sowing dates and expand its cultivation to new niches. However, in peas, HS tolerance strategies are mostly unclear, especially as it affects many developmental phases of plants when exposed to HS. While most of the information is derived from plants exposed to HS at reproductive phase under controlled environmental conditions, the knowledge under field conditions is limited due to complexities of exposure to stress without confounding effects of other climatic conditions. In this review we have comprehensively summarize the existing knowledge about the impact of HS on different economic traits in *Pisum* including physiological, biochemical, and molecular mechanisms operating under HS conditions. In addition, we discuss challenges, and breeding strategies for the development of HS resilient pea cultivars using conventional and molecular tools.

## 2 Impact of HS on peas

**Figure 1:**
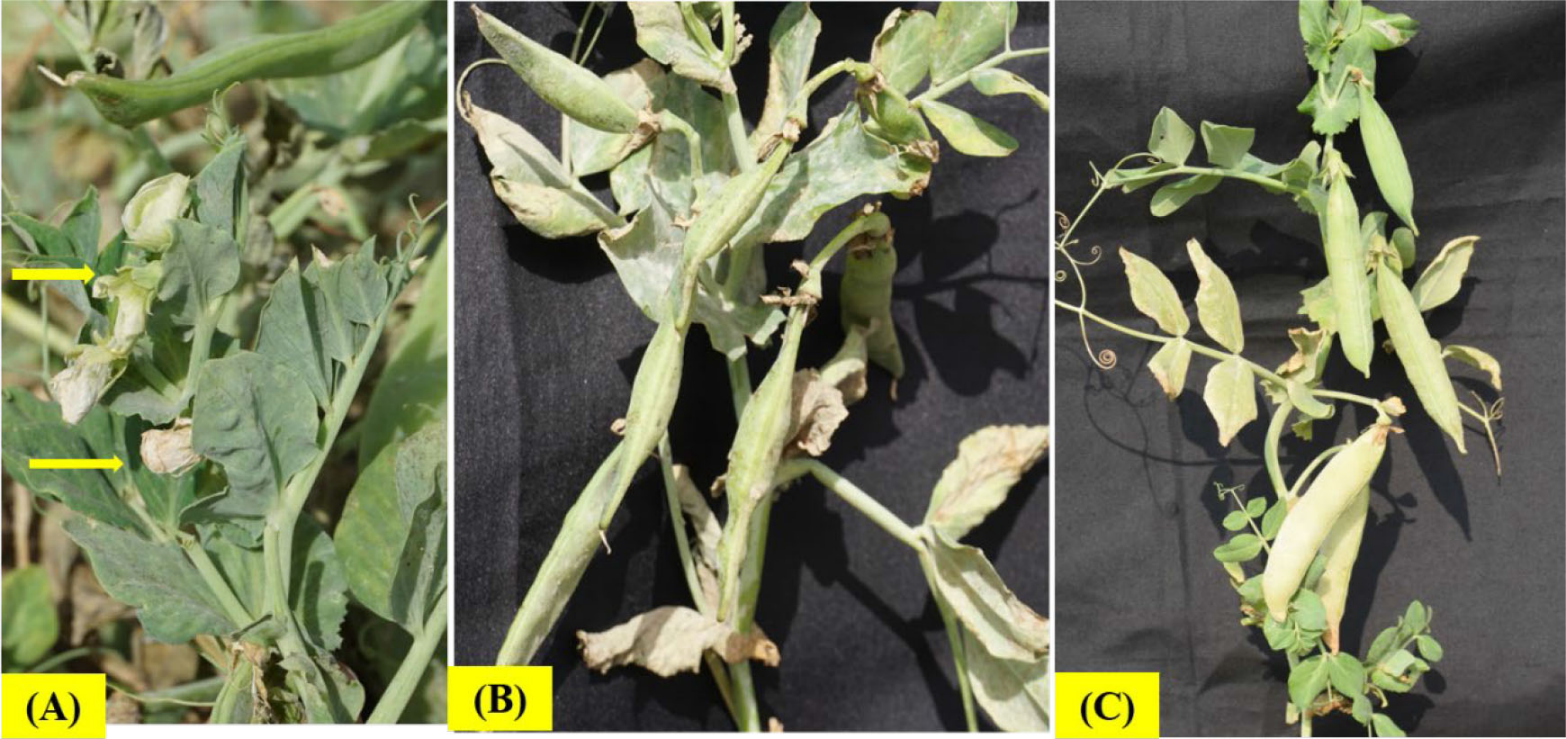
Genotypic differences for heat tolerance in vegetable peas at Indian Council of Agricultural Research (ICAR)-Indian Institute of Vegetable Research, Varanasi, India, showing heat sensitivity at temperature ≥32°C, **(A)** Effect of HS on floral parts; **(B)** Misshaped and unfilled pods in the heat sensitive genotype; **(C)** Duly filled pod formation in the heat tolerant genotype.

In peas, the HS could be sub-categorized into two phases, HS at vegetative stage and HS at reproductive stage. HS at vegetative phase is more challenging for the growers of vegetable peas, who prefers short duration picking types of peas preferably during September-October month of year (with prevailing temperature >30-32°C) in most of Asian countries (**Figure 1**). However, reproductive phase HS is important for field pea cultivars having longer growing duration wherein flowering usually coincides with higher temperatures during March and April months, especially in the Indian subcontinent. HS at early vegetative or reproductive growth stage decreases all the yield components as hot dry weather interferes with optimum plant growth, pollination and seed setting, thereby reduces the number of pods/plant and pod weight (Mohapatra et al., 2020; Tafesse et al., 2020). Furthermore, reproductive phase is more prone to the HS than the vegetative phase (Prasad et al., 2017; Lamichaney et al., 2021).

### 2.1 Impact on vegetative stage

**Figure 2:**
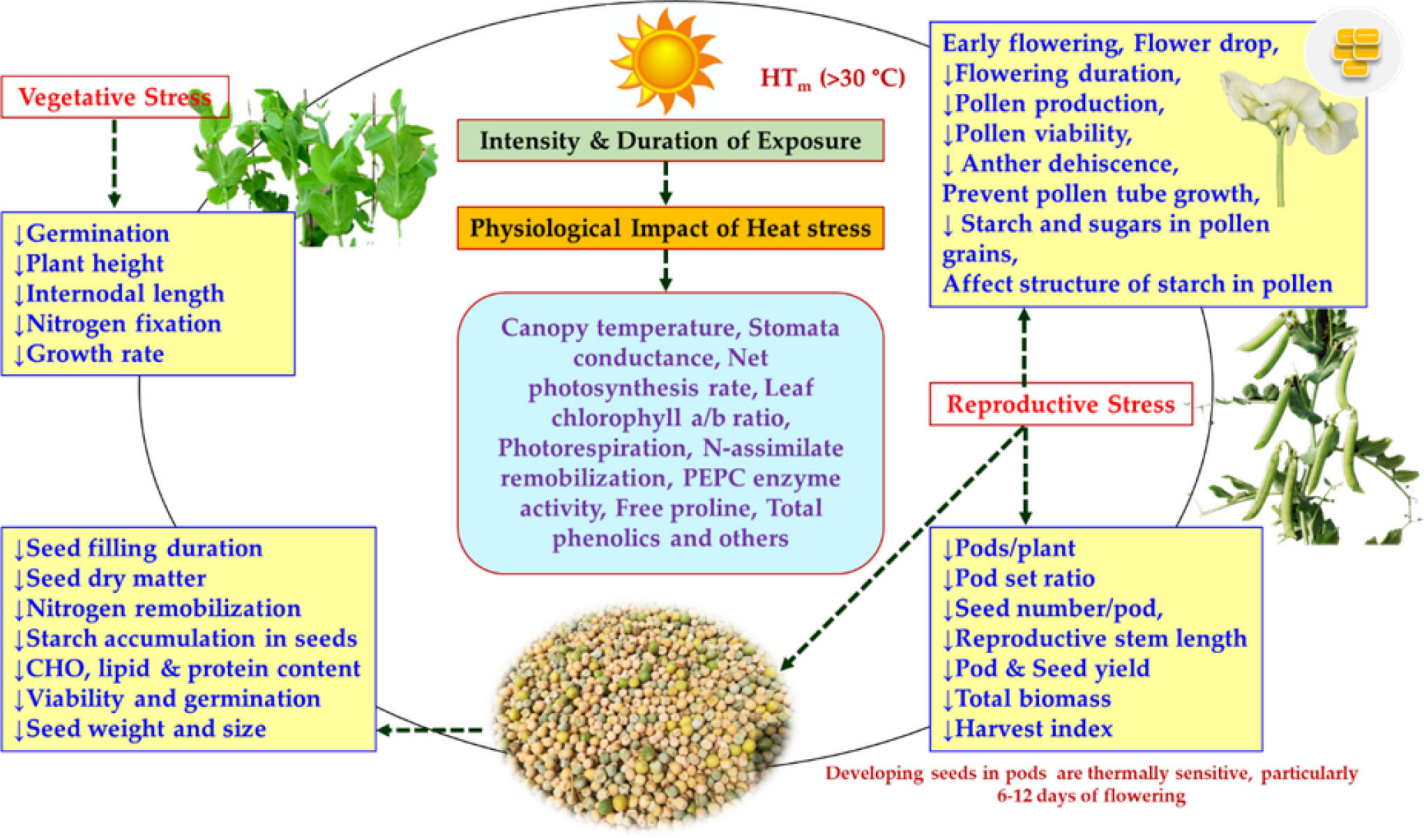
A diagram showing the physiological impact of HS in *Pisum* (Guilioni et al., 2003; Haldimann and Feller, 2005; Vijaylaxmi, 2013; Jiang et al., 2015; Larmure and Munier-Jolain, 2019; Mohapatra et al., 2020; Tafesse et al., 2020; Lamichaney et al., 2021).

The ideal temperature for vegetative growth in peas is 15–20°C (Mahoney, 1991) and the HS consequences are determined by intensity, duration and timing of heat exposure to the plant. HS has a significant impact on germination and vegetative growth of various legumes that includes reduction in shoot growth, root number, root diameter, reduced stomatal conductance and leaf water content, leaf curling, wilting and yellowing (Kaushal et al., 2013; Sita et al., 2017). The details of the HS impact on pea plant especially during vegetative growth phase is summarized in **Figure 2**. Seeds harvested from different HS conditions like HS-I (moderately late sown; November 30; T_MAX_=25.9 ± 0.11°C during flowering) and HS-II (very late sown; December 15; T_MAX_=30.6 ± 0.15°C during flowering) were noted with an average germination reduction of 4-8% in various cultivars (Lamichaney et al., 2021). The maximum impact was observed in late maturing cultivars (maturity >115 days) with germination loss of nearly 16% as compare to early genotypes (maturity <105 days) with nearly 4% loss. Further, Nemeskeri (2004) reported a day/night temperature of 30/30°C hampered the development of primary root in pea. The length of root in the small-seeded pea variety reduced by 69.3% when compared to the control (20/10°C), while a greater decline (73.8%) was recorded in the large-seeded pea varieties. High temperature (HTemp; 30/25°C) known to reduce leaf size and also promote early senescence of pea lower leaves (Munier-Jolain and Carrouée, 2010; Huang, 2016) with detrimental effect on leaf physiological functions (McDonald and Paulsen, 1997). Nodulation of pea plants is known to be adversely affected when pea plants are exposed to 30°C (Frings, 1976) along with reduction in plant height and biomass (Vijaylaxmi, 2013). Pea germplasm holds lot of phenotypic variation for leaves, canopy types and plant growth habit, thereby emphasis should be placed on identification of these traits that could have significant adaptive response under HS as an early first step to breed cultivars more resilient to HS. Similarly, further validation is also needed on role of root architecture system and canopy colour (pigmentation) under HS. Early and medium maturity group could perform better under HS conditions based on the timing of temperature stress conditions.

### 2.2 Impact on reproductive traits

HS has adverse effects on flowering and yield-related parameters during the reproductive period in peas (**Figure 2**). Mild HS (25-30°C) did not cause the abscission of reproductive organs, but it did cause the abortion of organs on higher nodes and does affect the seed filling inside the developing pods due to poor growth (Guilioni et al., 1997; Guilioni et al., 2003). In their early experiments on breeding for HS tolerance in peas, Lambert and Linck (1958) revealed that 6 h of HTemp (32°C) exposure for three or more days reduces seed yield in an indeterminate variety ‘Alaska’. Considerable impact was also recorded on the reproductive organs under HTemp (33/30°C; day/night) conditions with accelerated crop maturity (Guilioni et al., 1997). Seed development was temperature sensitive, as the exposure to 28-31°C for 6 h for 2-4 days, particularly after 6-12 days of flowering results in significant reduction in the total number of seeds/pod (Jeuffroy et al., 1990). Such reduction was also reported by other researchers (Karr et al., 1959; Stanfield et al., 1966; Nonnecke et al., 1971; Poggio et al., 2005).

Jiang et al. (2015) tested pea plant at 36/18°C day/night temperature for 7 days, and reported significant reduction in pollen germination (%), pollen tube length, seed number/pod and seed/ovule ratio over control plants at 24/18°C day/night temperature. Todorova et al. (2016) noted flower drop in peas when exposed to >30°C. Reduction in reproductive stem length, internode length, flowering duration, pod number, pod set ratio and seed yield was also documented under HS in peas (Tafesse, 2018; Jiang et al., 2020). Lamichaney et al. (2021) revealed that on an average 33% of ovules failed to set seeds in peas under late sowing conditions where maximum temperature during reproductive period was about 33°C. Similarly, exposure to 35/18 °C (day/night temperature) resulted in poor ovule and embryo sac expansion (Osorio et al., 2021). Additionally, a reduction in germination percentage was noted in the seeds of plants when exposed to HS (Lamichaney et al., 2021). In other legumes like lentil, day/night temperature at or above 35/20°C caused pod abortion, reduction in flower numbers, pollen viability, germination, stigmatic function, ovular viability, pollen tube elongation and shorter reproductive phase (Sita et al., 2017). Exposure of HTemp (32°C or above) for three or more days could negatively impact reproductive processes specifically on gamete formation and viability, fertilization, and seed setting leading to lower seed numbers in peas.

### 2.3 Percentage losses

HS causes severe yield losses by adversely affecting several traits in peas. When mean daily temperature was raised by nearly 2.2°C and 1.4°C, reduction has been reported for a number of traits like, water use efficiency (by 30.4% and 26.1%), duration of crop growth (by17 days and 10 days), yield (by 17.5% and 11.1%) and input/output ratio (by 1.20 and 1.11), respectively (Xiao et al., 2009). Similarly, a reduction in plant height (60.2%), total biomass yield (61.7%), seed yield (68.9%) and harvest index (19.3%) has also been observed (Vijaylaxmi, 2013). HS can increase the canopy temperature of pea plant from 24.9°C to 27.8°C which in turn affects other traits like reduction in the reproductive stem length (by 37%), flowering time (by 21%), pod quantity (by 30%), and seed production (by 16%) (Tafesse et al., 2019). The reduction in seed set (%) in HS-I (moderately late sown; November 30; T_MAX_= 25.9 ± 0.11°C during flowering) and HS-II (very late sown; December 15; T_MAX_= 30.6 ± 0.15°C during flowering) was recorded as 7-15% in early-maturing genotypes and 6-12% in late-maturing genotypes (Lamichaney et al., 2021). In addition, a reduction in 100-seed weight to the tune of 8-15% in early, and 4-17% in late maturing cultivars were also reported. The seeds harvested from heat stressed plants showed reduced germination (4-8%) over normal harvested plants. Maximum reduction in germination (>15%) was noted in the late maturing cultivars. Larmure and Munier-Jolain (2019) revealed that HTemp in peas decreases the seed-filling duration (by 0.8 day/°C), seed dry-matter and N accumulation rates (by 0.8 and 0.032 mg/seed/day/°C, respectively), and N remobilization from vegetative organs to seeds (by 0.053 mg/seed/day/°C).

## 3 Impact on physiological, biochemical traits and molecular changes

The physiological, biochemical and molecular changes associated with HS in a number of legumes have been reported (Wahid et al., 2007; Bita and Gerats, 2013; Bhandari et al., 2017; Liu et al., 2019). To combat HS and continue its life cycle, plants use various defense strategies including heat escape, avoidance, or tolerance mechanisms. Heat escape is a simple physiological adaptation, while tolerance to the heat are predominantly characterized by the differential expression of a number of genes including enhanced expression of certain heat shock proteins (HSPs) (Srikanthbabu et al., 2002). Further, the susceptibility to HS in plants varies with the stage of plant development (Wahid et al., 2007). Till now, limited studies have been reported pertaining to the HS in *Pisum* and the effect of HS on key physiological and biochemical traits has been summarized in **Table 1**, **Figure 2**.

**Table 1:** Effect of heat stress on key physiological, agronomical, and biochemical traits in *Pisum*.

Effects on Different Traits	References
Less nitrogen fixation in nodules	Frings, 1976
Less seeds per pod	Jeuffroy et al., 1990
Poor growth and more synthesis of *hsp18.1* and *hsp70* transcripts & HSP104 and HSP90 proteins	Srikanthbabu et al., 2002
Less seeds per plant and poor photosynthesis rate	Guilioni et al., 2003
Poor activity of phosphoenolpyruvate carboxylase (PEPC) enzyme	Chinthapalli, 2003
Lower net photosynthesis (*Pn*) and higher leaf temperature, photorespiration (*Pr*)	Haldimann and Feller, 2005
Less chlorophyll a, b and total carotenoid contents and more chlorophyll florescence ratio (F_690_/F_735_)	Georgieva and Lichtenthaler, 2006
Poor membrane stability index, plant height, total biomass yield, seed yield and harvest index	Vijaylaxmi, 2013
Poor growth and more heat shock protein synthesis *(Pshsp22.7*, *Pshsp22.9* and *Pshsp26.2)*	Talalaiev and Korduym, 2014
Poor percentage pollen germination, pollen tube length, pod length, seed number/pod, seed/ovule ratio, seed-weight, and size	Jiang et al., 2015; Jiang et al., 2017, Jiang et al., 2020
Decreased leaf pigments content and net photosynthesis rate	Todorova et al., 2016
Less free proline, total phenolics and hydrogen peroxide	Todorova et al., 2016
Poor plant growth and more activities of catalase, superoxide dismutase and guaiacol peroxidase	Todorova et al., 2016
Poor nitrogen fixation in nodules, N assimilate remobilization in plants and seeds	Larmure and Munier-Jolain, 2019
Less leaf chlorophyll a, chlorophyll b, and carotenoid concentrations, plant height, reproductive stem length, internode length, flowering duration, pod number, pod set ratio and seed yield. More canopy temperature (CT), leaf chlorophyll a/b ratio, leaf wax and leaf anthocyanin concentrations	Tafesse, 2018; Tafesse et al., 2019
Less number of pods and seeds/plant	Mohapatra et al., 2020
Poor seed germination, seed setting, seed yield, viability, and 100-seed weight	Lamichaney et al., 2021
More flower drop, shorter reproductive phase, reduced pod filling, abortion of seeds within pods and reduced yield	Susmita et al., 2020

### 3.1 Physiological traits

In peas, HS is known to reduce a number of physiological parameters like net photosynthetic rate (*Pn*) (Guilioni et al., 2003), overall N_2_ fixation and N assimilate remobilization (Larmure and Munier-Jolain, 2019). An increase in leaf temperature, photorespiration (*P_r_*) and healthy green appearance of young leaves over older leaves was recorded when plants were exposed to 32°C (compared to 25°C) (Haldimann and Feller, 2005). Leaf photosynthesis (*Pn*) started to decrease when leaf temperature touches ~30°C, while >80% reduction was recorded at 45°C. In addition, HS increases the canopy temperature (CT), leaf chlorophyll *a/b* ratio, leaf wax, leaf anthocyanin and reduces leaf chlorophyll *a*, chlorophyll *b*, and carotenoids in peas (Tafesse, 2018). In general, a cooler canopy is considered a desirable trait under HTemp, as is positively associated with high yield (Pradhan et al., 2014). Further, stomatal conductance directly affects transpirational cooling and expresses significant relationship between stomatal conductance and canopy temperature (Amani et al., 1996). Higher stomatal conductance and associated leaf cooling provides heat avoidance at HTemp (Xu et al., 2000). Traits such as stay-green and leaf waxiness show better adaptation under both HS and drought stress (DS) conditions (Xu et al., 2000; Kumar et al., 2010; Buschhaus and Jetter, 2011). HTemp (38°C) stress cause >20% decrease in leaf pigment content and significant suppression of net photosynthesis rate in pea (Todorova et al., 2016). HS also results in the depletion of sugar and starch contents not only in developing seeds, but also in pollen grains (Kaushal et al., 2013; Liu et al., 2013; Ruan, 2014; Liu et al., 2019). In pollen grains, the down regulation of hexose transporter negatively impacts the sugar transport leading to altered carbohydrate metabolism and starch deficiency (Jain et al., 2007). Thus, besides the agronomical traits, screening based on above physiological traits could lead to better understanding of heat tolerance mechanism. Additionally, these traits serve as indirect selection tools for improvement of HS tolerance.

### 3.2 Biochemical traits: role of ROS and phytohormones

At the cellular level, HS causes membrane protein denaturation, enzyme activation in mitochondria and chloroplasts, changes in membrane permeability and integrity, resulting in reduced ion flux, electrolyte leakage, changes in relative water content (RWC), toxic compound production, and a general disruption of homeostasis that reduces cell viability (Sita et al., 2017; Nijabat et al., 2020). HS impose oxidative stress to plant and provoke higher generation of Reactive Oxygen Species (ROS), including free radicals (O^•−^_2_ and OH^•^) and non-radicals (H_2_O_2_ and ^1^O_2_) mainly localized in the mitochondria, chloroplast and peroxisomes, with secondary sites in endoplasmic reticulum, cell wall, cell membrane and apoplast (Das and Roychoudhury, 2014; Medina et al., 2021). The excess production of ROS results in cellular damage that manifest as degradation of biomolecules including pigments, proteins, lipids, carbohydrates, and DNA (deoxyribonucleic acid), resulting in plant cellular death (Das and Roychoudhury, 2014). Impaired photosynthetic machinery during HS may be due to the inactivation of Rubisco and/or the associated enzymes. To ensure their survival, the antioxidant machinery activates as an inbuilt defense mechanism to cope with HS (Ding et al., 2016; Awasthi et al., 2017), although antioxidant quenching varies among the species and genotypes (Hasanuzzaman et al., 2013). The pea plants when exposed to HTemp (up to 38°C), a decrease in free proline, total phenolics, and hydrogen peroxide was observed, followed by an increase in catalase, superoxide dismutase, and guaiacol peroxidase activity (Todorova et al., 2016). Similarly, Kumar et al. (2013) observed higher suppression in antioxidant activity in sensitive chickpea genotypes over tolerant genotypes. Further, in peas, mitochondrial nucleoside diphosphate kinase (mtNDPK) enzyme was found to interact with a novel 86-kD protein, which is synthesized *de novo* in pea leaves upon exposure to heat (Escobar Galvis et al., 2001).

Phyto-hormones such as auxin, gibberellin (GA) and cytokinin (CK) are positively involve in regulating plant reproductive tolerance under HS (Ozga et al., 2016; Liu et al., 2019). Foliar application of auxins 4-chloroindole-3-acetic acid (4-Cl-IAA) at early reproductive stage of pea can increase the seed yield under HS (Abeysingha, 2015). Similarly, heat tolerant cultivars of common bean show lesser reduction of indole-3-acetic acid (IAA) content in flowers and pods than the sensitive cultivars resulting in lesser loss in pod and seed number under HS (Ofir et al., 1993). Among the other crop plants, Dobrá et al. (2015) observed a transient decrease in ABA and a small increase in cytokinin levels in *Arabidopsis* leaves during HS, which was further consistent with stimulation of transpiration as the prime cooling mechanism in leaves. Besides, ethylene hormone also play a negative role in legume reproduction under HS *via* induction of oxidative damage resulting in higher flower abscission and decreased pod set in soybean (*Glycine max* L.) (Djanaguiraman and Prasad, 2010; Djanaguiraman et al., 2011).Simultaneously, application of ethylene perception inhibitor 1-Methylcyclopropene is known to prevent reproduction failure by inhibiting ethylene production in soybean (Djanaguiraman and Prasad, 2010; Djanaguiraman et al., 2011). Although much information is available in other legumes, the adverse effects of HS at the cellular level such as impaired photosynthetic machinery, activation of various defense processes, and role of phytohormones, in peas is limited and requires in-depth investigation.

### 3.3 Synthesis of heat shock protein/factors

Heat shock proteins (HSPs) are evolutionarily conserved chaperones that prevent protein misfolding and denaturation induced by external stresses including HS (Will et al., 2017). First discovered in 1962 (Kregel, 2002), the regulation of HSPs/heat shock factors (HSFs) are known to govern HS tolerance in peas (Shah et al., 2020). Some plants synthesize up to 30-40 HSPs in response to HS (Mansfield and Key, 1987; Al-Whaibi, 2011) and it is assumed that the diversity of these proteins represents an adaptation to HS. The water-soluble nature of HSPs imparts heat tolerance through hydration of cellular structures. Different HSPs families have specific roles in mitigating the HS in plants. Broadly five HSPs are characterized in plants include, HSP20, HSP60, HSP70, HSP90 and HSP100. HSP20 helps in degradation of misfolded proteins, HSP60 and HSP70 are most commonly known and conserved heat shock compounds (Kültz, 2003). Wood et al. (1998) exposed peas plants to HTemp (37°C for 6 h) that accumulated two low molecular weight (LMW) HSPs (22 kDa). Talalaiev and Korduym (2014) determined the effects of temperature on the expression of HSPs of peas and conform extreme sensitivity of these genes to HS tolerance. Most of the highly induced genes include ER-localized *Pshsp22.7*, mitochondrial *Pshsp22.9* and chloroplast *Pshsp26.2* so that the expression of these genes increased up to several thousand-fold relative to the controlled seedlings at 42°C. Similarly, the induced seedlings accumulated higher levels of *hsp18.1* and *hsp70* transcripts as well as HSP104 and HSP90 proteins (Srikanthbabu et al., 2002). Mitochondrial nucleoside diphosphate kinase (*mtNDPK*) is reportedly involved in HS response through its interaction with a novel heat shock inducible 86-kD protein in peas (Escobar Galvis et al., 2001). Improved HS tolerance in peas was reported by incorporating ‘*HsfA1d*’ isolated from *Arabidopsis thaliana* (Shah et al., 2020). Recently, Huang et al. (2021a) have identified two ethylene response factors (*ERF_95_*, *ERF_97_*) enhancing HS tolerance in plants through *EIN3-ERF_95_*/*ERF_97_-HSFA2* transcriptional cascade that regulates a set of genes including heat responsive genes. Similarly, many heat stress-related proteins (Priya et al., 2019) and protein synthesis elongation factor EF-Tu (Ristic et al., 2008; Fu et al., 2012) play an important role in heat tolerance of plants

## 4 Screening environments

For breeding of the pea varieties having acquired thermotolerance, there is a need to identify the accurate screening environments and methods. Various controlled environments *viz.*, phytotrons, growth chambers, hydroponics, greenhouses along with natural screening in open field conditions or pots have been used in various crops (Sarsu et al., 2018; Balla et al., 2019; Lu et al., 2022). Comprehensive reviews are available summarizing various screening methods that are being used for the identification of thermotolerant genotypes through traits related to leaves (e.g., membrane thermostability, chlorophyll content, photosynthetic efficiency, chlorophyll fluorescence and stomata conductivity), flowers (e.g., pollen viability, pollen germination, fertilization and ovule viability), roots (e.g., depth, density and architecture), biomolecules (e.g., antioxidants, osmolytes, phytohormones, HSPs, and other stress proteins), and omics approaches (e.g., phenomics, transcriptomics and genomics) (Chaudhary et al., 2020).

### 4.1 Screening under field and controlled environments

Under field conditions, the strategy of growing plants with staggered sowing dates in anticipation of receiving HS at different stages has been used in many crops including pea (Jiang et al., 2017; Lamichaney et al., 2021). Simultaneously, such screening protocols are quite challenging due to heat escape or no guaranteed consistent HTemp conditions, interactions factors such as evaporative demand, wind, irrigation status, relative humidity, soil, cultural practices and other interactions and confounding effects. Moreover, field screening needs a thorough characterization of prevailing temperature at different growth stage of plants, preferably with a known thermotolerant check (Ayenan et al., 2019). Therefore, precise phenotyping under natural conditions through integration of modern phenomics tools could lead to increased breeding efficiency *via* large scale screening of germplasm with more accuracy and efficiency (Pratap et al., 2019).

Few better HS screening approaches has been developed like screening under phytotron, growth chambers, and greenhouses with the advantage of controlled growth conditions including temperature. However, such facility required huge investments, with insufficient space for screening large populations. Further, standardization of lethal temperature is important in case of controlled screening. Under natural growing conditions, plants get exposed to stress gradually known as induction stress (IS), rather than the severe stress (SS) at lethal temperature. Studies have shown that plants showed greater survival to IS than SS as many stress signaling pathways get triggered with the expression of stress responsive genes in IS (Srikanthbabu et al., 2002). Therefore, it is advisable that before screening of genotypes for thermotolerance, it is better to expose them to IS before their final exposure to SS (Ayenan et al., 2019). In an experiment, Verma et al. (2019) reported a temperature of 43°C for 3 h as lethal for survival of ‘Azad pea-1’ seedlings in peas, and they used this screening protocol to identify the heat tolerant pea genotypes. In recent past, temperature induction response (TIR) has been utilized to screen the pea genotypes for thermotolerance (Srikanthbabu et al., 2002; Verma et al., 2019). TIR is a method in which seedlings are subjected to an induction temperature for optimal expression of stress genes before being exposed to an extremely HTemp, which is otherwise lethal to non-induced seedlings. HS tolerance can be assessed using various viability assays, visual assessment, and testing under hotspot locations (Govindaraj et al., 2018). Thus, combination of field based screening protocols followed by their validation under controlled environmental conditions or vice versa would be a reliable approach for evaluation of heat tolerance or susceptibility.

### 4.2 HS threshold temperature (T_max_) in *Pisum*

The temperature at which seed germination, seedling and vegetative development, flowering, fruit set, and fruit ripening are seriously affected is referred to as the upper threshold temperature (Wahid et al., 2007). While the sensitivity to HS in peas has been intensively studied and published since early 1950s, still the threshold temperatures (T_max_) for yield reduction have been inconsistently reported. Various researchers have suggested different temperature range beyond which peas yield is reduced significantly. Lambert and Linck (1958) considered a temperature of 32°C is much more detrimental in yield reduction of peas than 27°C and 29°C. Nonnecke et al. (1971) reported continued exposure at 27/17°C (day/night temperature) resulting in significant yield loss. Jiang et al. (2015) indicated 36°C as the critical temperature for a significant reduction in pollen germination and pollen tube length. He explained that the actual threshold temperature for HS in field is hard to deduce and interpret, because irrigation increases the threshold by several degrees. Similarly, a few other studies suggested 25.6°C (Pumphrey and Ramig, 1990), 31°C (Jeuffroy et al., 1990), 25°C (Sadras et al., 2013) and 28°C (Bueckert et al., 2015) as a maximum threshold temperature in peas. Even, yield reduction is reported to decline at 16°C and above (Stanfield et al., 1966), which may not be true for the all the cultivars. Further, some researchers believed that night temperature has more critical role (Karr et al., 1959), while others advocate the importance of diurnal mean temperature as a better predictor of pea plants response to HTemp (Stanfield et al., 1966).

## 5 Traditional breeding for HS in *Pisum*

### 5.1 Harnessing crop germplasm repertoire

Screening of crop gene pool and landraces for yield and HS tolerance in a targeted environment is a simple approach to identify HS tolerant genotypes in peas (**Table 2**), with considerable genetic variations within cultivated types. Further, crops wild species have been successfully utilized in pre-breeding program for development of HS in various crops such as rice (*Oryza sativa* L.) (Mammadov et al., 2018), pigeon pea (*Cajanus cajan* L.) (Ramakrishna et al., 2021) and wheat (*Triticum aestivum* L.) (Ali et al., 2010). To the best of our knowledge, till now there are no reports available on the use of wild *Pisum* species for the transfer of HS tolerance in cultivated genotypes. Intensive screening is needed to scan the available wild pea genetic resources (primary and secondary gene pool) for the novel variations for HS tolerance which could be utilized to broaden the gene pools. Furthermore, local pea land races have reported to carry many important traits for various biotic and abiotic stresses including HS and such races should be utilized in pea breeding program aimed to improve the HS tolerance (Bahuguna et al., 2015; Kilasi et al., 2018). In China, Wang et al. (2022) screened 2358 worldwide pea accessions for three years and identified 26 extremely heat tolerant accessions. These accessions can be used for breeding for HS tolerance in pea. In India, some local pea land races are being grown by various farming communities which are more tolerant to HS e.g., *Kasmiria*, *Shihara* local (VRPSel-1), and *Magadi Local*. Sometimes heat adaptive traits are also associated with a certain undesirable traits in peas and identified local races were found to possess smaller pod size, lesser grain number and reduced yield (Devi et al., 2018; Susmita et al., 2020). Additionally, the ‘semi leafless (*afila*)’ types which is a heat responsive trait is found more commonly in pulse type genotypes and is linked with late flowering and podding traits. Thus, this trait mostly got ignored when bred for vegetable-pea type cultivars. Checa et al. (2020) devised a rapid breeding method for the introgression of recessive *afila* gene into commercial cultivars by using them as a recurrent parents (RP) through backcross breeding programs. Furthermore, the other traits like higher pod number, more reproductive nodes and longer flowering duration are common in many field peas genotypes. But such traits now should be introgressed into vegetable type with no undesirable linkages through repeated backcrossing. Recently Devi et al. (2018); Devi et al. (2021) also reported a high yielding, multi-flowered genotypes of vegetable peas attributed mainly due to higher pod number and longer flowering duration. The inheritance of these traits must be worked out and accordingly more precise breeding strategies should be opted for development of suitable cultivars. India has a large collection of pea germplasm (4680; http://www.nbpgr.ernet.in/) in the national gene bank which needs to be systematically evaluated against the HTemp and HS tolerant accessions could be identified for further use in identification of genes and breeding programs.

**Table 2:** List of pea genotypes identified for heat tolerant and their associated traits.

Genotypes	Screening Method	Stage	Responsive Traits/Parameters	Types	Country	Reference
Acc.623 and Acc.765	TIR	Vegetative stage	Recovery growth, Enhances expression of *hsp_s_*	Pulse type	India	Srikanthbabu et al., 2002
PFD 99-7, IPFD 3-17, IPFD 2-6, IPFD 1-10, HUDP 16 and DPR 13	Field trials	Reproductive stage	Membrane stability index at podding, plant height, biological yield, seed yield and harvest index	Pulse type	India	Vijaylaxmi, 2013
Arka Uttam, Arka Apoorva, IIHR 544, IIHR 13-1, IIHR 680, PMR 37, Swarna Mukti, KTP 4 and VRPMR 11	TIR	Vegetative stage	Recovery growth	Vegetable type	India	Verma et al., 2019
JP-625, IARI-2877, PMR-38 II, EC318760, EC-328758 and IARI-2904	Polyhouse	Reproductive stage	Pod setting, pods/plant, seeds/plant, seed size and weight	Pulse type	India	Mohapatra et al., 2020
40-10, Naparnyk and CDC Meadow	Growth chamber and Field trials	Reproductive stage	Ovules and seeds/pod	Pulse type	Canada	Jiang et al., 2020
Arka Uttam, Arka Chaitra, and Arka Tapas, Magadi local* (Land race)	Field trials	Reproductive stage	Pod weight, pods/plant, seed/pod, and yield	Vegetable type	India	Susmita et al., 2020

TIR, temperature induction response; * Magadi Local: heat tolerant land race reported from southern India.

### 5.2 Identification of traits associated with HS adaptation in *Pisum*

Proper screening methods and identification of most responsive traits that adapt better to elevated temperature are key component of breeding for HS tolerance. Mohapatra et al. (2020) reported that pods/plant in HS tolerant genotypes vary from 15-45; seeds/plant from 35-197; 25 seed-weight from 3.5 to 6.7 g and seed diameter from 53-80 mm. A highly positive correlation between number of seeds/plant with number of pods/plant; seed diameter and seed-weight, whereas negative correlation between seed-weight and pods/plant in the heat tolerant pea genotypes were identified under HS conditions. Further allocation of photosynthetic products to enhance seed weight resulted in reduced number of pods and seeds/plant among heat tolerant pea genotypes. Importance of canopy based traits in heat adaption, adding that pea cultivars with the semi leafless type (carrying *Afila* gene), upright growing nature, resistance to lodging were better adapted to heat stressed environments than cultivars with the normal leaf and vining habit. Such cultivars are characterized by less surface area and lower transpirational water loss (Tafesse, 2018; Tafesse et al., 2019).

In addition, they could maintain cooler canopy temperature through upright growth. Although semi-leafless plant types have been identified as excellent genotype for improved production and lodging resistance in peas (Singh and Srivastava, 2015). But, Mohapatra et al. (2020) observed that this may and may not be absolutely true, as some of the semi-leafless genotypes (e.g. VL-40, KPMR-615, DDR-61, KPMR-557) were grouped under heat susceptible category while others in heat tolerant category (e.g. HUDP-25, IPF-400, HFP-4, DDR-56). Further, late flower termination and high pod number/plant were found promising and helpful indices for high yield potential under warmer environments (Huang et al., 2017). Similarly, Jiang et al. (2020) proposed that to maintain or improve yield performance in a warming climate, new cultivars need to produce more reproductive nodes and abort fewer pods/plant and fewer seeds/pod. He further explained that cultivars with a lower 1000 seed-weight retained more ovules and seeds/pod than large-seeded cultivars. In addition, canopy hue has been found associated with leaf pigments and radiation reflection that may have a crucial role in physiological/biochemical protection from vital plant processes. Similarly, leaf surface wax is found positively correlated with water band index, thus maintaining the cooler canopy temperature. However, rigorous studies are needed to explore this basic breeding features further. Direct selection for traits positively associated with HS tolerance such as number of pods per plant, number of seeds per pod, seed weight, seed diameter, canopy temperature, leaf morphology, greater reproductive nodes, partitioning to seeds, and yield should be kept in mind when selecting genotypes for HS tolerance.

## 6 Genomics for HS in *Pisum*

### 6.1 QTL mapping for HS traits

*Pisum* being a model plant, used extensively at phenotypic and molecular level and its genome sequence got released in 2019 (Kreplak et al., 2019). However, very little progress has been made in term of underlying molecular mechanism (at genomic level) for HS in peas as compared to other winter season legumes like chickpeas and lentil. Tafesse et al. (2020) evaluated 135 accessions of peas in five environments for 10 HS responsive traits using GWAS (Genome Wide Association Studies) and identified 32 associated markers and 48 candidates genes for heat tolerance in pea (**Table 3**). Similarly, in the same GWAS population QTLs related to heat and drought stresses were identified for traits like lamina wax, petiole wax, stem thickness, flowering duration, normalized difference vegetation index (NDVI) and normalized pigment and chlorophyll index (NPCI) (Tafesse et al., 2021). QTL (quantitative trait loci) mapping to HS tolerance have been done in other legume crops such as chickpea (Paul et al., 2018). Similarly, in cowpea, QTLs for pod number per peduncle and two genes for HS tolerance were mapped (Lucas et al., 2013; Pottorff et al., 2014). Even though pea is an important crop, only limited studies have been conducted to identify genomic regions associated with HS tolerance, therefore more efforts are required to use the available molecular resources for conducting the mapping and tagging of genes.

**Table 3:** QTLs discovery of heat responsive traits with their genomic locations and candidate genes in *Pisum*.

Traits	Loci (No.)	Genomic location	Variance explained (PVE%)	Gene ID	Reference
SPAD value/Chlorophyll concentration	06	LGIII	7-13	*Psat5g221440, Psat5g224400, Psat5g224360, Psat5g224280, Psat5g299080, Psat5g299040, Psat5g301440, Psat5g301400, Psat5g303880*, *Psat5g303840*, *Psat5g303800* and *Psat5g303760*	Tafesse et al., 2020
Photochemical reflectance index	02	LGII and LGVII	9	*Psat6g234040, Psat6g234000* and *Psat7g148080*	Tafesse et al., 2020
Canopy temperature	02	LGIII and LGIV,	6	*Psat4g203800, Psat4g203760, Psat5g169800* and *Psat5g169760*	Tafesse et al., 2020
Reproductive stem length	07	LGV LGIV LGIII and LGVII	4-6	*Psat3g006600, Psat3g006560, Psat4g020520, Psat5g299080, Psat5g299040, Psat5g303680, Psat7g013080, Psat7g013040, Psat7g015240, Psat7g015200, Psat7g015160, Psat7g057080* and *Psat7g057040*	Tafesse et al., 2020
Pod number	09	LGI, LGV, LGIII and one locus on non‐chromosomal scaffold	7-10	*Psat2g060680, Psat2g144160, Psat2g155280, Psat2g157440, Psat2g166600, Psat2g166560, Psat2g166520, Psat2g005000, Psat2g004960, Psat3g111000, Psat3g110960* and *Psat5g270480*	Tafesse et al., 2020
Internode length	06	LGIV LGIII LGII and LGVII	6-7	*Psat4g039600, Psat4g047680, Psat4g047640, Psat4g047600, Psat5g299080, Psat5g299040, Psat6g211160, Psat7g120120*	Tafesse et al., 2020
Lamina Wax	04	LGVI LGIV LGII and LG7	–	*Psat1g139360, Psat4g112480 and Psat7g076840*	Tafesse et al., 2021
Petiole Wax	03	LGIV LGVII Uscaffold03717_87257	–	*Psat4g011120, Psat7g186040, Psat0s3717* *g0080*	Tafesse et al., 2021
Stem thickness	03	LGVII LGII and Uscaffold03985_59708	–	*Psat7g071920, Psat7g072040, Psat7g208760, Psat0s3985* *g0040*	Tafesse et al., 2021
Flowering duration	02	LGV and LGIII	–	*Psat3g006600, Psat5g140600*	Tafesse et al., 2021
Normalized difference vegetation index (NDVI)	01	LGII	–	*Psat6g028080, Psat6g028120*	Tafesse et al., 2021
Normalized pigment and chlorophyll index (NPCI)	02	LGIII and LGII	–	*Psat5g299040, Psat6g231000*	Tafesse et al., 2021

Many reports have identified the most responsive traits for HS, and genomic locations/genes responsible for these traits through number of linkage studies (Jiang et al., 2020; Mohapatra et al., 2020; Tafesse et al., 2020; Lamichaney et al., 2021) under normal growing conditions *viz*., plant height (Irzykowska and Wolko, 2002; Tar’an et al., 2003; Hamon et al., 2013; Ferrari et al., 2016; Gali et al., 2019); lodging resistance (Tar’an et al., 2003; Jha et al., 2017; Gali et al., 2019); seed-weight, number and yield (Timmerman-Vaughan et al., 2005); days to flowering (Huang et al., 2017; Gali et al., 2019), pod number and seed-weight (Huang et al., 2017); shorter internodes (Weeden, 2007) and grain yield (Tar’an et al., 2004; Krajewski et al., 2012; Gali et al., 2019). Further, significant progress has been made towards the discovery of genes, and associated/flanking markers for these traits (Dirlewanger et al., 1994; Zhang et al., 2006; Tayeh et al., 2015; Gali et al., 2019). Thus, this information can be utilized and further validated for the presence of any QTL(s) expressing itself over the varied agroecology with greater adaptation under the HS conditions.

### 6.2 Candidate genes and transcription factors for HS

The huge data generated through NGS (next generation sequencing) can be associated to the putative candidate genes responsible for the HS tolerance in pea. RNA sequencing has been done in many legumes for understanding the genetic factors governing HS related traits. In pea, based on the gene ontology several candidate genes have been identified that could be associated with the HS tolerance (Tafesse et al., 2020; Tafesse et al., 2021). The constitutively expressed and tissue specific genes are summarized in **Tables 3** and **4**. The functional annotation of these genes will benefit to understand their role in HS tolerance. The transcriptome profiling of a heat tolerant line ‘PR11-2’ and ‘CDC Amarillo’ was conducted under HS at 38°C for 3 h and from the heat stressed anthers and stipules they could identify 588 and 879 differentially expressed genes (DEGs), respectively (Huang et al., 2021b). The major DEGs were found related to the cell wall macromolecule metabolism, lipid transport, lipid localization and lipido-metabolic processes. Heat stress leads to rapid lipid remodeling in the leaves, pollen, and developing seeds due to GDSL lipase activity. This will have drastic effect on yield and other nutritional parameters (Huang et al., 2021b). Suppressing or over-expressing any of the lipase genes that are differentially regulated during HS will have positive effect on stress tolerance. Thus, HS response was found variety specific and biological processes like cellular response to DNA damage stimulus in stipule, electron transport chain in anthers were observed in heat tolerant lines (Huang et al., 2021b). The biological processes related to cell wall were found significantly downregulated when exposed to HS, which could be the reason of cell was damage under HS. In the anther of cultivar ‘PR11-2’ the upregulated biological processes belonged to respiratory electron transport chain, lignin catabolic process and cellular modified amino acid catabolic process.

**Table 4:** The candidate genes identified in pea under the heat stress conditions.

Trait	Gene ID	Protein Name	Gene Ontology	Reference
Chlorophyll index (SPAD value)	*Psat5g221440*	Amidohydrolase like protein	Hydrolase activity, acting on carbon-nitrogen (but not peptide) bonds	Tafesse et al., 2020
	*Psat5g224400*	Cysteine-rich receptor-like protein kinase 25	Integral component of membrane; ATP binding; protein kinase activity	
	*Psat5g224360 Psat5g224280*	Pentatricopeptide repeat-containing protein at1g11290-like protein	Zinc ion binding	
	*Psat5g299080*	Kinesin-related protein 4-like	–	
	*Psat5g299040*	PPR containing plant-like protein (Putative tetratricopeptide-like helical domain-containing protein)	–	
	*Psat5g301440*	Embryo-specific 3	–	
	*Psat5g301400*	Nuclear pore protein	Membrane; nuclear pore; structural constituent of nuclear pore; mRNA transport; protein transport	
	*Psat5g303880*	Putative sterile alpha motif/pointed domain-containing protein (SAM domain protein)	Negative regulation of transcription, DNA-templated	
	*Psat5g303840*	Gamma-glutamylcyclotransferase At3g02910	Gamma-glutamylaminecyclotransferase activity. transferase activity	
	*Psat5g303800*	Nuclear fusion defective 4	Integral component of membrane	
Photochemical reflective index	*Psat6g234040*	Putative GTP 3, 8-cyclase	Mo-molybdopterin cofactor biosynthetic process	
	*Psat6g234000*	Riboflavin biosynthesis protein ribF	FMN adenylyltransferase activity; riboflavin biosynthetic process	Tafesse et al., 2020
	*Psat7g148080*	TATA-binding-like protein	ATP binding	
Canopy temperature	*Psat4g203800*	Ethylene-responsive transcription factor-like protein At4g13040	Nucleus; DNA binding; DNA-binding transcription factor activity	Tafesse et al., 2020
	*Psat5g169800*	ABC transporter C family member 3-like isoform X1	Integral component of membrane; ATP binding; ATPase activity, coupled to transmembrane movement of substances	
	*Psat5g169760*	Retrovirus-related Pol polyprotein from transposon TNT 1-94	Retrotransposon nucleocapsid; nucleic acid binding; DNA integration	
Reproductive stem length	*Psat3g006600*	Uncharacterized protein LOC101515092	Integral component of membrane	Tafesse et al., 2020
	*Psat3g006560*	L-allo-threonine aldolase-like protein (Putative aldehyde-lyase)	Lyase activity; cellular amino acid metabolic process	
	*Psat4g020520*	Alkaline-phosphatase-like protein (Putative Type I phosphodiesterase/nucleotidepyrophosphatase/phosphate transferase)	Integral component of membrane; mannose-ethanolamine phosphotransferase activity; GPI anchor biosynthetic process	
	*Psat5g299080*	Kinesin-related protein 4-like	–	
	*Psat5g299040*	PPR containing plant-like protein (Putative tetratricopeptide-like helical domain-containing protein)	–	
	*Psat5g303680*	Putative sterile alpha motif/pointed domain-containing protein (SAM domain protein)	–	
	*Psat7g013080*	aldehyde dehydrogenase family 2-member C4-like	Oxidoreductase activity, acting on the aldehyde or oxo group of donors, NAD or NADP as acceptor	
	*Psat7g013040*	Cst complex subunit ctc1-like protein	Telomere maintenance	
	*Psat7g015240*	Ribosomal L7Ae/L30e/S12e/Gadd45 family protein	–	
	*Psat7g015200*	Tesmin/TSO1-like CXC domain protein	–	
	*Psat7g057080; Psat7g057040*	tRNA (Cytosine (34)-C (5)) methyltransferase-like protein	RNA binding; tRNA (cytosine-5-) methyltransferase activity	
Internodal length	*Psat4g039600*	Eukaryotic translation initiation factor 3 subunit C (eIF3c) (Eukaryotic translation initiation factor 3 subunit 8) (eIF3 p110)	Eukaryotic 43S preinitiation complex; eukaryotic 48S preinitiation complex; eukaryotic translation initiation factor 3 complex; translation initiation factor activity; translation initiation factor binding; formation of cytoplasmic translation initiation complex	Tafesse et al., 2020
	*Psat4g047640*	Ras GTPase-activating protein-binding protein 1-like	RNA binding	
	*Psat5g299080*	Kinesin-related protein 4-like	–	
	*Psat5g299040*	PPR containing plant-like protein (Putative tetratricopeptide-like helical domain-containing protein)	–	
	*Psat6g211160*	Transmembrane amino acid transporter family protein	Integral component of membrane	
Pod number	*Psat2g144160*	Pectin acetylesterase	Cell wall; extracellular region; integral component of membrane; hydrolase activity; cell wall organization	Tafesse et al., 2020
	*Psat2g155280*	60S ribosomal protein l8-like	ribosome; structural constituent of ribosome; translation	
	*Psat2g157440*	Putative ATPase, AAA-type, core, AAA-type ATPase domain-containing protein (p-loop nucleoside triphosphate hydrolase superfamily protein)	ATP binding; hydrolase activity	
	*Psat2g166600*	Probable serine/threonine-protein kinase At1g01540 isoform X1	Integral component of membrane; ATP binding; protein kinase activity	
	*Psat2g166560*	PI-PLC X domain-containing protein At5g67130	Phosphoric diester hydrolase activity; lipid metabolic process	
	*Psat2g005000*	Nup133/Nup155-like nucleoporin	Structural constituent of nuclear pore	
	*Psat2g004960*	Cation-transporting ATPase plant (Putative calcium-transporting ATPase)	Integral component of membrane; nucleotide binding	
	*Psat3g111000*	Phosphomannomutase	Cytoplasm; phosphomannomutase activity; GDP-mannose biosynthetic process	
	*Psat3g110960*	bifunctional protein FolD 4, chloroplastic	Methylenetetrahydrofolate dehydrogenase (NADP	
	*Psat5g270480*	Heat shock protein 70 (HSP70)-interacting protein, putative	–	
Lamina wax	*Psat1g139360*	Hydrolase activity + hydrolysing O-glycosyl compounds	–	Tafesse et al., 2021
	*Psat4g112480*	Arp2/3 complex + 34 kD subunit p34-Arc	Actin filament binding; structural constituent of cytoskeleton	
	*Psat7g076840*	NnrU protein	Isomerase activity	
Petiole wax	*Psat4g011120*	Aminotransferase class-III	Adenosylmethionine8-amino-7oxononanoate transaminase activity; dethiobiotin synthase activity; pyridoxal phosphate binding	Tafesse et al., 2021
	*Psat7g186040*	Pyridine nucleotide disulphide oxidoreductase	Oxidoreductase activity	
Stem thickness	*Psat0s3985g0040*	Myb/SANT-like DNA-binding domain	–	Tafesse et al., 2021
Flowering duration	*Psat5g140600*	SWIB/MDM2 domain	–	Tafesse et al., 2021
Normalized difference vegetation index (NDVI)	*Psat6g028080*	PB1 domain	Calcium ion binding	Tafesse et al., 2021
	*Psat6g028120*	Protein kinase domain	ATP binding; protein serine/threonine kinase activity	
Normalized pigment and chlorophyll index (NPCI)	*Psat5g299040*	PPR repeat family		
	*Psat6g231000*	Dual specificity phosphatase + catalytic domain	Protein tyrosine/serine/threonine phosphatase activity	Tafesse et al., 2021

In cowpea cDNA-AFLP (complementary DNA-amplified fragment length polymorphism) was used to understand the expression of various thermo- tolerant genes (Simões-Araújo et al., 2002). HSFs were studied in legumes like soybean and Medicago (Kotak et al., 2007). In soybean, the role of HSP20 and GmHsfA1in relation to HS have been evaluated (Chen, 2006; Zhu et al., 2006; Lopes-Caitar et al., 2013). The heat shock transcription factors (HsTFs) mediate the activation of heat-responsive genes and one such factor that have been identified to have a major role in stress tolerance is WRKY transcription factors (TFs) (Chen et al., 2012). *Arabidopsis thaliana* HsFs is a typical representative of plant HsFs having a modular structure (Baniwal et al., 2007). In a transformation study, the pea plant transformed with *Arabidopsis*’s heat shock factor ‘*HsF1d*’ showed improved ROS scavenging system to confront the HS (Shah et al., 2020). The HS tolerance in the transgenics is due to increased antioxidant enzyme activity and reduced hydrogen peroxide. Other HsFs derived from *Arabidopsis* have proven their worth in HS tolerance in rice (Zhang et al., 2013) and wheat (Xue et al., 2014), these factors can also be tried in the HS studies in pea. Several HSF has also been studied and identified in the chickpea such as CarHSFA2, A6 and B2 which were upregulated and has importance in the regulatory network related to HS (Chidambaranathan et al., 2018). Transcription factors aid in regulation of the genes and control their expression. There is a need for identification and validation of these transcription factors in pea and the identified factors should be compared with other legumes to gain a clear understanding about their role in HS tolerance.

In pea the gene discovery is limited to finding of HSP genes. Among the different HSP genes reported in pea, the expression of *PsHSP18.1* and *PsHSP71.2* genes appeared to be heat inducible (DeRocher et al., 1991; DeRocher and Vierling, 1995). *PsHSP 18.1* was in the cytoplasm, whereas *PsHSP21* and *PsHSP22* were located in chloroplasts and mitochondria, respectively. The relation of HSPs to heat tolerance was subsequently confirmed as the induction of these HSP genes improved survival rate of pea seedlings and mature plants at HTemp (Srikanthbabu et al., 2002). Moreover, several HSP genes had greater heat-induced expression in a heat tolerant cultivar, Acc.623, than in the susceptible genotype Acc.476 (Srikanthbabu et al., 2002). The transcription levels of cytoplasmic HSPs got increased after the HS in the pea (Huang et al., 2021b). The *HSP70* homologues were constitutively expressed in pea after HS. The HSP70 proteins are ATP driven molecular chaperons encoded to target different cellular compartment like mitochondria, chloroplast, endoplasmic reticulum, and the cytoplasm (Huang et al., 2021b). These putative genes and proteins need further validation to exactly pinpoint the role of each and every gene and protein in governing the HS.

### 6.3 Genetic engineering for achieving HS

Breeding transgenics is an alternate strategy for the development of HS tolerant cultivars in pea. The low variation for HS tolerance in pea can be addressed through introgression of foreign gene from related or unrelated organism by genetic engineering. Till date, only one study is known for the development of transgenics in pea. However, success in development of transgenics for HS tolerance has been demonstrated in wheat, rice, maize and other crops (Guo et al., 2016; Ni et al., 2018; El-Esawi et al., 2019). In pea the HS tolerance was achieved through incorporation of HSF (*HsfA1d*) isolated from *Arabidopsis thaliana* using agrobacterium mediated transformation (Shah et al., 2020). In the transformed plants five-fold increase in the expression of *HsfA1d* was observed under the HS condition. In the transformed plants upon HS significant increase in SOD activity, proline content and ascorbate peroxidase activity were observed. These enzymes function as antioxidant and decrease the hydrogen peroxide activity thereby improving the tolerance against the HS in pea. Several other genes have been characterized in Arabidopsis, rice, wheat and maize which can be utilized in the development of genetically modified pea for achieving HS (Yadav et al., 2022).

Due to regulatory hurdles the transgenic breeding approaches has not been widely used. Under such a scenario the CRISPR/Cas9 technology is gaining traction in crop breeding and genetic improvement of many targeted traits including abiotic stress tolerance in many crop species (Li et al., 2022a). However, there is limited research on peas and other legumes which need attention. Recently, Agrobacterium mediated transformation system of hairy roots was developed and gene *phytoene desaturase* (*PsPDS*) causing albinism was edited in pea (Li et al., 2022b).

## 7 Breeding approaches

Different breeding methods that can be used in pea to improve the HS tolerance, the option includes germplasm selection, pure line selection, pedigree breeding and backcross breeding. As a general rule, all breeding methods suitable for breeding of self-pollinated crops are equally applicable to peas. The highly self-pollinated nature of pea facilitates the easy development of pure lines that can be established through identifying genetic resources with heat tolerant attributes. While screening, distinction must be made between thermotolerance nature *vs* growth potential, as plant with more growth, in general, grow better in wide environmental conditions (Wahid et al., 2007). Further, the developed pure lines can be used in breeding programs such as pedigree breeding, back cross breeding, and recurrent selection. The developed pure line can also be used to map QTL(s) associated with the complex traits such as HS and yield in the HS during vegetative and reproductive stages. These pure lines can also be used to study the inheritance of the HS tolerance trait and for crop improvement by combining with other traits of interest. At HS, the breeding method can be designed to select for a higher number of flower production and pod setting. Efficient selection technique during the breeding program is crucial for identification of HS tolerant parental lines, inheritance studies and utilization through breeding programs.

The direct selection for traits such as photosynthetic rate and reproductive fitness can be one of the ways for identification of HS tolerant genotypes (Prasad et al., 2008); for example, during cowpea breeding for HS tolerance the selection was done for genotypes with abundant flower and pod production (Marfo and Hall, 1992), resulting in the development of HS tolerant cowpea variety California Blackeye 27 (CB27) (Ehlers et al., 2000). The varieties of common bean (CIAT, 2006)and chickpea (Gaur et al., 2019)were developed through germplasm screening and selection. In the case of vegetable pea, three heat tolerant cultivars namely Arka Uttam, Arka Chaitra and Arka Tapas were developed for cultivation during the off seasons (Susmita et al., 2020). In wheat, the physiological breeding was proposed to combine set of physiological traits for genetic effect on yield (Cossani and Reynolds, 2012). This method can be applied to pea crop breeding for improvement of HS tolerance. Indirect selection of secondary traits with high heritability can be used to improve the high yield under HS condition. In case of maize indirect selection for secondary traits resulted in the development of two maize genotypes, VL05728 and VL05799 for better seed setting during HS (Alam et al., 2017).Traditional breeding clubbed with MAS can improve selection efficiency, reduce the time and increase the confidence about the identified genes/QTLs. The few QTLs/genes for HS can be pyramided as was done in the case of rice (Kilasi et al., 2018).Genomic selection, genome wide association studies (GWAS) and marker assisted recurrent selection (MARS) are other available options for efficient development of pea against HS.

## 8 Way forward

### 8.1 Appropriate screening methodology

**Figure 3:**
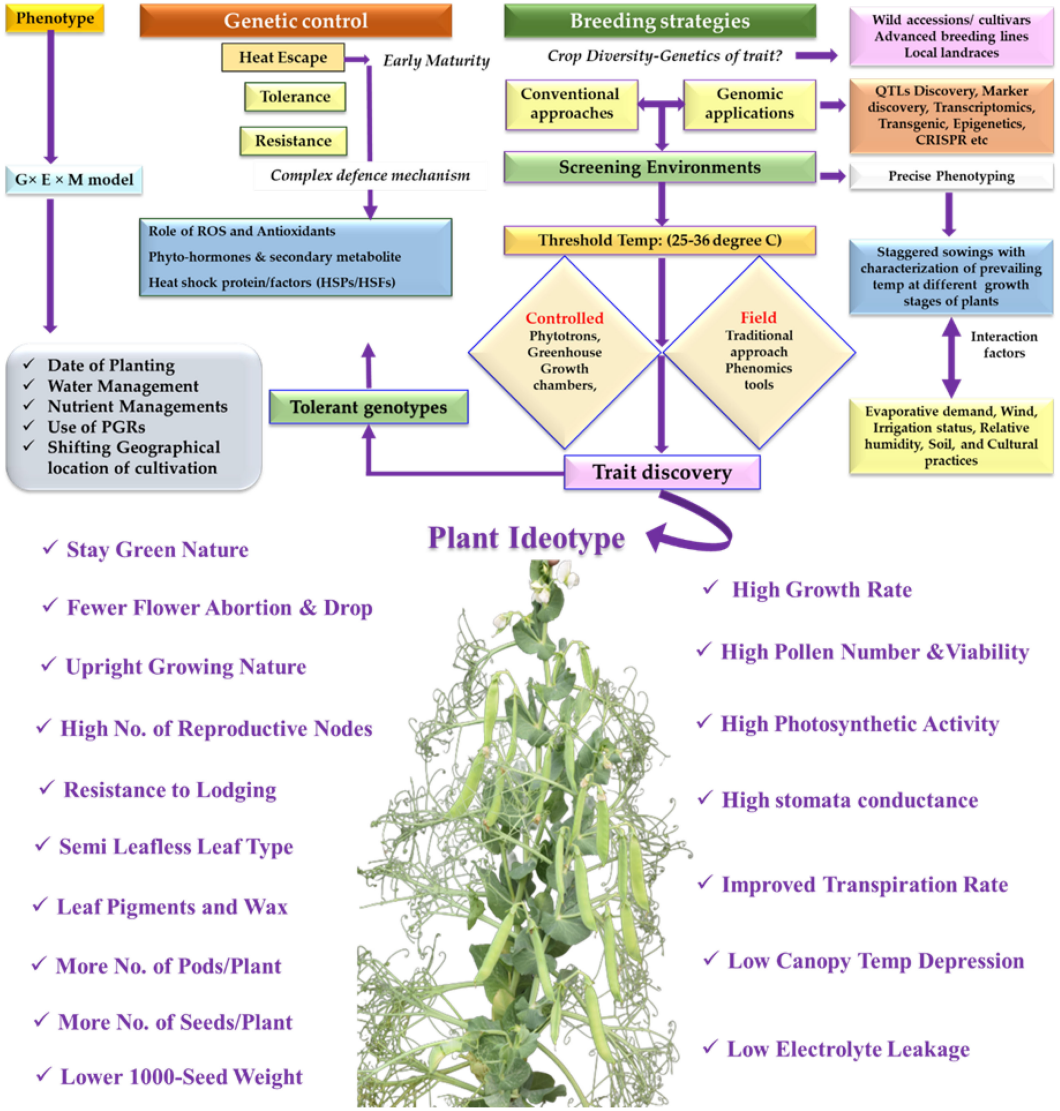
Breeding for heat stress tolerance in *Pisum;* The phenotypic performance of cultivars under heat stress is determined by Genotype × Environment × Management Model. Ideotype breeding for HS includes combination of Agro-morphological and physiological traits.

HS tolerance can be improved through conventional as well as genomic approaches (**Figure 3**). However, these approaches are time consuming and expensive (Khan et al., 2020). Further, varied maturity groups in peas (early, mid, and late) and end use grouping (vegetable types and pulse types), complicates the screening process. As stated, HS at vegetative stage is important in peas when cultivars are being bred for September and October maturity (extra early) groups under Asian conditions. On contrary, breeding vegetable peas for late sown conditions (during March and April) or for pulse type, the HS is mostly experienced at the reproductive phase. Moreover, early flowering genotypes escape HS due to their early maturity. For robust screening, long HS imposition must be followed by screening the genotypes for HS tolerance from seedling to maturity or exposing plant at specific growth stages depending upon the local or regional environmental conditions based on when HS occurs under field conditions. Use of phenomics tools is important to screen the large set germplasm with more precision to evaluate the complex adaptive traits such as plant architecture, physiological traits and other quantitative parameters (Pratap et al., 2019). Further, there is a need to incorporate physiological screening protocols rather than over emphasizing on the yield and agronomical traits, as these show proximity with the markers with considerable level of variability and heritability. Some such traits include selection based upon pollen viability, canopy temperature depression (CTD), electrolyte leakage, membrane stability, chlorophyll fluorescence or photosynthetic function and green leaf area duration.

### 8.2 Trait discovery, genetics, and molecular breeding

Identification of traits in peas controlling any adaptive response of cultivars to HS is an important first step for the effective breeding for the HS tolerant cultivars. In past, most of the HS related studies in peas were focused on the reproductive stages (Guilioni et al., 1997; Guilioni et al., 2003; Jiang et al., 2015) and minimal efforts have been devoted for the identification of potential traits at vegetative stage including canopy-based tolerance and their relations to reproductive tolerance. Further, the genetics of these traits should also be precisely carried out under the HS conditions as many HS governing traits responded differentially e.g. yield associated traits have been reported with low heritability response in tomato under HS conditions (Hanson et al., 2002). There are only a few reports in *Pisum* which highlight the quantitative inheritance of few heat-responsive traits (**Tables 3**, **4**), and these still need further validation. Although many genomic studies have identified some QTLs/genes for certain agronomical and quality traits under normal growing environment in *Pisum*, still there is a need to develop mapping populations for identification of heat responsive QTLs under HS environments.

### 8.3 Managing and regulating stress as short-term strategy

Since the well-established breeding strategies for HS tolerance is time consuming and costly; thus, the growing environment can be modified for short term gains through use of plant growth regulators, biofertilizers, irrigation management, and nutrient management as reported in other crops. Further, growing short-duration cultivars and altering the planting date before the onset of HS during critical growth stages of the crop might be advantageous. It is one of the practices done by few vegetables growers from Indo-Gangetic regions of India (Varanasi), who grows short duration varieties like Kashi Udai and Kashi Nandini, sowing is done by Mid-January and picking is ready by mid of March (60-65 days) before the onset of HTemp.

### 8.4 Use of plant growth regulators and biofertilizers

The endogenous plant defense system can be boosted through the use of plant growth regulating chemicals such as polyamines having free radicle scavenging features and antioxidant activities (Groppa and Benavides, 2008; Gill and Tuteja, 2010). By spraying the plants with spermine, the adverse physiological consequences of HS could be reduced in peas (Todorova et al., 2016). Furthermore, increasing literature on use of plant growth promoting endophytic bacteria (PGPEB) as an alternative, environmentally friendly strategy towards boosting of the crop production by reducing the adverse consequences of HS on crops such as sorghum (*Sorghum bicolor* L. Moench) (Ali et al., 2009), chickpea (Srivastava et al., 2008), wheat (Ali et al., 2010), tomato (*Solanum lycopersicum* L.) (Issa et al., 2018), soybean (Khan et al., 2020), and potato (*Solanum tuberosum* L.) (Bensalim et al., 1998) provides new options for pea.

### 8.5 Alleviation of HS by nutrient management

Better plant nutrition can successfully mitigate an array of adverse effects of HTemp stress. The use of macronutrients such as K, Ca and micronutrients such as B, Se and Mn under HS can help to activate the metabolic and biological processes that help to maintain the high water potential of tissues and therefore increase the HS tolerance (Waraich et al., 2012). The application of plant nutrient like N, K, Ca, and Mg has also been found to reduce toxicity to ROS by increased the amount of antioxidant enzymes such as superoxide dismutase (SOD). However, there is a paucity of information dedicated to the nutritional dynamics, specifically, on micronutrient-use efficiency under climatic changes, which influences crop nutrient absorption, transport, and remobilization in *Pisum*. More studies should be done aiming to understand the nutritional dynamics of peas under HS conditions.

## 9 Conclusions

Peas being cool season crop have a narrow window of its cultivation. There is high demand for the varieties which can be successfully cultivated in the non-traditional areas to increase in overall area, cultivation, and production. Its cultivation and area expansion are challenged by the projected rise in temperatures both seasonal means and occurrence of extreme temperature events. Though, a few reports of heat tolerant pea genotypes are available, yet identification of more HS tolerant genotypes through controlled and field studies are needed. In addition, this should be well integrated with high-throughput phenotyping platforms available in various pea cultivating countries. Prolong HS imposition from seedling to maturity or at specific growth stages based on the occurrence in the region need to be followed while screening the material for HS tolerance. This should be integrated with the physiological based interventions and germplasm characterization for yield. The pea ideotype for warmer regions must carry certain traits such as, semi leaflessness with upright growing habit, lodging tolerance, more reproductive nodes, pods/plant, ovules/pod, increased seed numbers and higher 1000 seed-weight (**Figure 3**). Physiologically, the pea genotypes should have high growth rate, higher gamete (pollen and ovule) viability, seed-set, photosynthetic activity, improved transpiration rate, low canopy temperature depression (CTD), and less membrane damage.

Plant genetic architecture and correlation of these traits needs to be established to understand their differential response under HS. Further, as plant phenotype is known to be influenced by genotype, environment and genotypic × environmental interactions. In addition, cultural management practices (M) are often included as third separate factor for better crop yield, leading to need to understand the G× E ×M interactions and models (Driedonks et al., 2016) for better adaption. One can attain a greater yield by modifying any of these factors, such as genotype, environment, and crop management (e.g., crop duration, phenology, environmental conditions, soil type, sowing date, irrigation, nutrient management). However, planting the tolerant genotype is the most feasible alternative for pea cultivation in warmer climates. The HS can have detrimental impacts on nutritional quality (Sehgal et al., 2018), most likely owing to a lack of assimilates and reduced nutrient remobilization. For example, lycopene content in tomato (Stevens and Rudich, 1978; Alsamir et al., 2021), tocopherols in rice (Britz et al., 2007), storage proteins and amino acids in lentil (Sita et al., 2018; Sehgal et al., 2019) and micronutrients (Hein et al., 2022), sugars (Shah and Paulsen, 2003) and proteins (Zhang et al., 2018) in wheat. However, such quality and nutrition-related gaps in legume crops including peas are limited and must be understood to quantify impact on nutritional value.

## Author contributions

Conceptualization, JD, VS, and GM; writing original draft and resources, JD, VS, GM, PJ, NG, RD, PS, TB, and PP. All authors contributed to the article and approved the submitted version.

## References

[B1] Abeysingha G. (2015). “The effects of auxins on seed yield parameters in wheat, pea and canola grown under controlled environmental and Western Canadian field conditions,” in MSc Thesis (Edmonton: University of Alberta.), 2015.

[B2] Alam M. A. Seetharam K. Zaidi P. H. Dinesh A. Vinayan M. T. Nath U. K. (2017). Dissecting heat stress tolerance in tropical maize (*Zea mays* l.). F. Crop Res. 204, 110–119. doi: 10.1016/j.fcr.2017.01.006

[B3] Ali M. B. Ibrahim A. M. H. Hays D. B. Ristic Z. Fu J. (2010). Wild tetraploid wheat (*Triticum turgidum* l.) response to heat stress. J. Crop Improv. 24, 228–243. doi: 10.1080/15427528.2010.481523

[B4] Ali S. Z. Sandhya V. Grover M. Kishore N. Rao L. V. V. B. (2009). *Pseudomonas* sp. strain AKM-P6 enhances tolerance of sorghum seedlings to elevated temperatures. Biol. Fertil Soils. 46, 45–55. doi: 10.1007/s00374-009-0404-9

[B5] Alsamir M. Mahmood T. Trethowan R. Ahmad N. (2021). An overview of heat stress in tomato (*Solanum lycopersicum* l.). Saudi J. Biol. Sci. 28, 1654–1663. doi: 10.1016/j.sjbs.2020.11.088 33732051 PMC7938145

[B6] Al-Whaibi M. H. (2011). Plant heat-shock proteins: A mini review. J. King Saud Univ. - Sci. 23, 139–150. doi: 10.1016/j.jksus.2010.06.022

[B7] Amani I. Fischer R. A. Reynolds M. P. (1996). Canopy temperature depression association with yield of irrigated spring wheat cultivars in a hot climate. J. Agron. Crop Sci. 176, 119–129. doi: 10.1111/j.1439-037X.1996.tb00454.x

[B8] Awasthi R. Gaur P. Turner N. C. Vadez V. Siddique K. H. M. Nayyar H. (2017). Effects of individual and combined heat and drought stress during seed filling on the oxidative metabolism and yield of chickpea (*Cicer arietinum*) genotypes differing in heat and drought tolerance. Crop Pasture Sci. 68, 823. doi: 10.1071/CP17028

[B9] Ayenan M. A. T. Danquah A. Hanson P. Ampomah-Dwamena C. Sodedji F. A. K. Asante I. K. . (2019). Accelerating breeding for heat tolerance in tomato (*Solanum lycopersicum* l.): an integrated approach. Agronomy 9, 720. doi: 10.3390/agronomy9110720

[B10] Bahuguna R. N. Jha J. Pal M. Shah D. Lawas L. M. Khetarpal S. . (2015). Physiological and biochemical characterization of NERICA-L-44: a novel source of heat tolerance at the vegetative and reproductive stages in rice. Physiol. Plant 154, 543–559. doi: 10.1111/ppl.12299 25302555

[B11] Balla K. Karsai I. Bónis P. Kiss T. Berki Z. Horváth Á. . (2019). Heat stress responses in a large set of winter wheat cultivars (*Triticum aestivum* l.) depend on the timing and duration of stress. PLoS One 14, e0222639. doi: 10.1371/journal.pone.0222639 31539409 PMC6754161

[B12] Baniwal S. K. Chan K. Y. Scharf K.-D. Nover L. (2007). Role of heat stress transcription factor hsfa5 as specific repressor of HsfA4. J. Biol. Chem. 282, 3605–3613. doi: 10.1074/jbc.M609545200 17150959

[B13] Bensalim S. Nowak J. Asiedu S. K. (1998). A plant growth promoting rhizobacterium and temperature effects on performance of 18 clones of potato. Am. J. Potato Res. 75, 145–152. doi: 10.1007/BF02895849

[B14] Bhandari K. Sharma K. D. Hanumantha Rao B. Siddique K. H. M. Gaur P. Agrawal S. K. . (2017). Temperature sensitivity of food legumes: a physiological insight. Acta Physiol. Plant 39, 68. doi: 10.1007/s11738-017-2361-5

[B15] Bita C. E. Gerats T. (2013). Plant tolerance to high temperature in a changing environment: scientific fundamentals and production of heat stress-tolerant crops. Front. Plant Sci. 4. doi: 10.3389/fpls.2013.00273 PMC3728475 23914193

[B16] Britz S. J. Prasad P. V. V. Moreau R. A. Allen L. H. Kremer D. F. Boote K. J. (2007). Influence of growth temperature on the amounts of tocopherols, tocotrienols, and γ-oryzanol in brown rice. J. Agric. Food Chem. 55, 7559–7565. doi: 10.1021/jf0637729 17725318

[B17] Bueckert R. A. Wagenhoffer S. Hnatowich G. Warkentin T. D. (2015). Effect of heat and precipitation on pea yield and reproductive performance in the field. Can. J. Plant Sci. 95, 629–639. doi: 10.4141/cjps-2014-342

[B18] Buschhaus C. Jetter R. (2011). Composition differences between epicuticular and intracuticular wax substructures: How do plants seal their epidermal surfaces? J. Exp. Bot. 62, 841–853. doi: 10.1093/jxb/erq366 21193581

[B19] Chaudhary S. Devi P. Bhardwaj A. Jha U. C. Sharma K. D. Prasad P. V. V. . (2020). Identification and characterization of contrasting genotypes/cultivars for developing heat tolerance in agricultural crops: current status and prospects. Front. Plant Sci. 11. doi: 10.3389/fpls.2020.587264 PMC7642017 33193540

[B20] Checa O. Rodriguez M. Wu X. Blair M. (2020). Introgression of the afila gene into climbing garden pea (*Pisum sativum* l.). Agronomy 10 1537. doi: 10.3390/agronomy10101537

[B21] Chen X. J. (2006). Cloning of GmHSFA1 gene and its overexpression leading to en-hancement of heat tolerance in transgenic soybean. Hereditas 11, 1411–1420. doi: 10.1360/yc-006-1411 17098711

[B22] Chen L. Song Y. Li S. Zhang L. Zou C. Yu D. (2012). The role of WRKY transcription factors in plant abiotic stresses. Biochim. Biophys. Acta - Gene Regul. Mech. 1819, 120–128. doi: 10.1016/j.bbagrm.2011.09.002 21964328

[B23] Chidambaranathan P. Jagannadham P. T. K. Satheesh V. Kohli D. Basavarajappa S. H. Chellapilla B. . (2018). Genome-wide analysis identifies chickpea (*Cicer arietinum*) heat stress transcription factors (Hsfs) responsive to heat stress at the pod development stage. J. Plant Res. 131, 525–542. doi: 10.1007/s10265-017-0948-y 28474118

[B24] Chinthapalli B. (2003). Dramatic difference in the responses of phosphoenolpyruvate carboxylase to temperature in leaves of C3 and C4 plants. J. Exp. Bot. 54, 707–714. doi: 10.1093/jxb/erg078 12554714

[B25] CIAT (2006) Annual report of the international center for tropical agriculture (CIAT). Available at: https://cgspace.cgiar.org/bitstream/handle/10568/73453/CIAT_Annual_Report_2015-2016_Synthesis.pdf?sequence=6.

[B26] Cossani C. M. Reynolds M. P. (2012). Physiological traits for improving heat tolerance in wheat. Plant Physiol. 160, 1710–1718. doi: 10.1104/pp.112.207753 23054564 PMC3510104

[B27] Das K. Roychoudhury A. (2014). Reactive oxygen species (ROS) and response of antioxidants as ROS-scavengers during environmental stress in plants. Front. Environ. Sci. 2. doi: 10.3389/fenvs.2014.00053

[B28] DeRocher A. E. Helm K. W. Lauzon L. M. Vierling E. (1991). Expression of a conserved family of cytoplasmic low molecular weight heat shock proteins during heat stress and recovery. Plant Physiol. 96, 1038–1047. doi: 10.1104/pp.96.4.1038 16668295 PMC1080890

[B29] DeRocher A. Vierling E. (1995). Cytoplasmic HSP70 homologues of pea: differential expression in vegetative and embryonic organs. Plant Mol. Biol. 27, 441–456. doi: 10.1007/BF00019312 7894010

[B30] Devi J. Dubey R. K. Mishra G. P. Sagar V. Verma R. K. Singh P. M. . (2021). Inheritance and stability studies of multi–flowering trait in vegetable pea (*Pisum sativum* l.), and its contribution in yield improvement. Sci. Hortic. (Amsterdam). 287, 110235. doi: 10.1016/j.scienta.2021.110235

[B31] Devi J. Mishra G. P. Sanwal S. K. Dubey R. K. Singh P. M. Singh B. (2018). Development and characterization of penta-flowering and triple-flowering genotypes in garden pea (*Pisum sativum* l. var. *hortense*). PLoS One 13, e0201235. doi: 10.1371/journal.pone.0201235 30059526 PMC6066227

[B32] Ding X. Jiang Y. He L. Zhou Q. Yu J. Hui D. . (2016). Exogenous glutathione improves high root-zone temperature tolerance by modulating photosynthesis, antioxidant and osmolytes systems in cucumber seedlings. Sci. Rep. 6, 35424. doi: 10.1038/srep35424 27752105 PMC5067582

[B33] Dirlewanger E. Isaac P. G. Ranade S. Belajouza M. Cousin R. de Vienne D. (1994). Restriction fragment length polymorphism analysis of loci associated with disease resistance genes and developmental traits in *Pisum sativum* l. Theor. Appl. Genet. 88, 17–27. doi: 10.1007/BF00222388 24185876

[B34] Djanaguiraman M. Prasad P. V. V. (2010). Ethylene production under high temperature stress causes premature leaf senescence in soybean. Funct. Plant Biol. 37, 1071–1084. doi: 10.1071/FP10089

[B35] Djanaguiraman M. Prasad P. V. V. Al-Khatib K. (2011). Ethylene perception inhibitor 1-MCP decreases oxidative damage of leaves through enhanced antioxidant defense mechanisms in soybean plants grown under high temperature stress. Environ. Exp. Bot. 71, 215–223. doi: 10.1016/j.envexpbot.2010.12.006

[B36] Dobrá J. Černý M. Štorchová H. Dobrev P. Skalák J. Jedelský P. L. . (2015). The impact of heat stress targeting on the hormonal and transcriptomic response in *Arabidopsis* . Plant Sci. 231, 52–61. doi: 10.1016/j.plantsci.2014.11.005 25575991

[B37] Driedonks N. Rieu I. Vriezen W. H. (2016). Breeding for plant heat tolerance at vegetative and reproductive stages. Plant Reprod. 29, 67–79. doi: 10.1007/s00497-016-0275-9 26874710 PMC4909801

[B38] Ehlers J. D. Hall A. E. Patel P. N. Roberts P. A. Matthews W. C. (2000). Registration of ‘California blackeye 27’ cowpea. Crop Sci. 40, 854–885.

[B39] El-Esawi M. A. Al-Ghamdi A. A. Ali H. M. Ahmad M. (2019). Overexpression of *AtWRKY30* transcription factor enhances heat and drought stress tolerance in wheat (*Triticum aestivum* l.). Genes (Basel). 10, 163. doi: 10.3390/genes10020163 30791662 PMC6410048

[B40] Escobar Galvis M. L. Marttila S. Håkansson G. Forsberg J. Knorpp C. (2001). Heat stress response in pea involves interaction of mitochondrial nucleoside diphosphate kinase with a novel 86-kilodalton protein. Plant Physiol. 126, 69–77. doi: 10.1104/pp.126.1.69 11351071 PMC102282

[B41] FAOSTAT (2019) 2018 Food and agriculture data. Available at: http://www.fao.org/faostat/en/#home (Accessed 10th October, 2020).

[B42] Ferrari B. Romani M. Aubert G. Boucherot K. Burstin J. Pecetti L. . (2016). Association of SNP markers with agronomic and quality traits of field pea in Italy. Czech J. Genet. Plant Breed. 52, 83–93. doi: 10.17221/22/2016-CJGPB

[B43] Frings J. F. J. (1976). The rhizobium-pea symbiosis as affected by high temperatures (Veenman: 89, Laboratorium voor Microbiologie).

[B44] Fu J. Momcilovic I. Prasad P. V. V. (2012). Roles of protein synthesis elongation factor EF-tu in heat tolerance in plants. J. Bot. 835836. doi: 10.1155/2012/835836

[B45] Gali K. K. Sackville A. Tafesse E. G. Lachagari V. B. R. McPhee K. Hybl M. . (2019). Genome-wide association mapping for agronomic and seed quality traits of field pea (*Pisum sativum* l.). Front. Plant Sci. 10. doi: 10.3389/fpls.2019.01538 PMC6888555 31850030

[B46] Gaur P. M. Samineni S. Thudi M. Tripathi S. Sajja S. B. Jayalakshmi V. . (2019). Integrated breeding approaches for improving drought and heat adaptation in chickpea (*Cicer arietinum* l.). Plant Breed. 138, 389–400. doi: 10.1111/pbr.12641

[B47] Georgieva K. Lichtenthaler H. K. (2006). Photosynthetic response of different pea cultivars to low and high temperature treatments. Photosynthetica 44, 569–578. doi: 10.1007/s11099-006-0073-y

[B48] Gill S. S. Tuteja N. (2010). Polyamines and abiotic stress tolerance in plants. Plant Signal. Behav. 5, 26–33. doi: 10.4161/psb.5.1.10291 20592804 PMC2835953

[B49] Govindaraj M. Pattanashetti S. K. Patne N. Kanatti A. A. (2018). “Breeding cultivars for heat stress tolerance in staple food crops,” in Next generation plant breeding Yelda Çiftçi. London: IntechOpen. doi: 10.5772/intechopen.76480

[B50] Groppa M. D. Benavides M. P. (2008). Polyamines and abiotic stress: recent advances. Amino Acids 34, 35–45. doi: 10.1007/s00726-007-0501-8 17356805

[B51] Guilioni L. Wéry J. Lecoeur J. (2003). High temperature and water deficit may reduce seed number in field pea purely by decreasing plant growth rate. Funct. Plant Biol. 30, 1151. doi: 10.1071/FP03105 32689097

[B52] Guilioni L. Wery J. Tardieu F. (1997). Heat stress-induced abortion of buds and flowers in pea: is sensitivity linked to organ age or to relations between reproductive organs? Ann. Bot. 80, 159–168. doi: 10.1006/anbo.1997.0425

[B53] Guo M. Liu J.-H. Ma X. Luo D.-X. Gong Z.-H. Lu M.-H. (2016). The plant heat stress transcription factors (HSFs): structure, regulation, and function in response to abiotic stresses. Front. Plant Sci. 7. doi: 10.3389/fpls.2016.00114 PMC4746267 26904076

[B54] Haldimann P. Feller U. (2005). Growth at moderately elevated temperature alters the physiological response of the photosynthetic apparatus to heat stress in pea (*Pisum sativum* l.) leaves. Plant Cell Environ. 28, 302–317. doi: 10.1111/j.1365-3040.2005.01289.x

[B55] Hamon C. Coyne C. J. McGee R. J. Lesné A. Esnault R. Mangin P. . (2013). QTL meta-analysis provides a comprehensive view of loci controlling partial resistance to *Aphanomyces euteiches* in four sources of resistance in pea. BMC Plant Biol. 13, 45. doi: 10.1186/1471-2229-13-45 23497245 PMC3680057

[B56] Hanson P. M. Chen J. Kuo G. (2002). Gene action and heritability of high-temperature fruit set in tomato line CL5915. HortScience 37, 172–175. doi: 10.21273/HORTSCI.37.1.172

[B57] Hasanuzzaman M. Nahar K. Alam M. Roychowdhury R. Fujita M. (2013). Physiological, biochemical, and molecular mechanisms of heat stress tolerance in plants. Int. J. Mol. Sci. 14, 9643–9684. doi: 10.3390/ijms14059643 23644891 PMC3676804

[B58] Hein N. T. Impa S. M. Wagner D. Bheemanahalli R. Kumar R. Tiwari M. . (2022). Grain micronutrient composition and yield components in field-grown wheat are negatively impacted by high night-time temperature. Cereal Chem. 99, 615–624. doi: 10.1002/cche.10523

[B59] Huang S. (2016). “Characterization of a pea recombinant inbred population for resistance to heat at flowering,” in M.Sc thesis (Saskatoon: University of Saskatchewan Saskatoon).

[B60] Huang S. Gali K. K. Lachagari R. V. B. Chakravartty N. Bueckert R. A. Tar’an B. . (2021b). Identification of heat responsive genes in pea stipules and anthers through transcriptional profiling. PLoS One 16, e0251167. doi: 10.1371/journal.pone.0251167 34735457 PMC8568175

[B61] Huang S. Gali K. K. Tar’an B. Warkentin T. D. Bueckert R. A. (2017). Pea phenology: crop potential in a warming environment. Crop Sci. 57, 1540–1551. doi: 10.2135/cropsci2016.12.0974

[B62] Huang J. Zhao X. Bürger M. Wang Y. Chory J. (2021a). Two interacting ethylene response factors regulate heat stress response. Plant Cell 33, 338–357. doi: 10.1093/plcell/koaa026 33793870 PMC8136883

[B63] Irzykowska L. Wolko B. S. W. (2002). Interval mapping of QTLs controlling some morphological traits in pea. Cell Mol. Biol. Lett. 7, 417–422. 12378244

[B64] Issa A. Esmaeel Q. Sanchez L. Courteaux B. Guise J.-F. Gibon Y. . (2018). Impacts of *Paraburkholderia phytofirmans* strain PsJN on tomato (*Lycopersicon esculentum* l.) under high temperature. Front. Plant Sci. 9. doi: 10.3389/fpls.2018.01397 PMC6201190 30405648

[B65] Jain M. Prasad P. V. V. Boote K. J. Hartwell A. L. Chourey P. S. (2007). Effects of season-long high temperature growth conditions on sugar-to-starch metabolism in developing microspores of grain sorghum (*Sorghum bicolor* l. moench). Planta 227, 67–79. doi: 10.1007/s00425-007-0595-y 17680267

[B66] Janni M. Gullì M. Maestri E. Marmiroli M. Valliyodan B. Nguyen H. T. . (2020). Molecular and genetic bases of heat stress responses in crop plants and breeding for increased resilience and productivity. J. Exp. Bot. 71, 3780–3802. doi: 10.1093/jxb/eraa034 31970395 PMC7316970

[B67] Jeuffroy Mh Duthion C. Meynard Jm P. A. (1990). Effect of a short period of high day temperatures during flowering on the seed number per pod of pea (*Pisum sativum* l). Agron. EDP Sci. 10, 139–145.

[B68] Jha A. B. Gali K. K. Tar’an B. Warkentin T. D. (2017). Fine mapping of QTLs for ascochyta blight resistance in pea using heterogeneous inbred families. Front. Plant Sci. 8. doi: 10.3389/fpls.2017.00765 PMC5422545 28536597

[B69] Jiang Y. Bueckert R. Warkentin T. Davis A. (2017). High temperature effects on in vitro pollen germination and seed set in field pea. Can. J. Plant Sci. 98, 71–80. doi: 10.1139/CJPS-2017-0073

[B70] Jiang Y. Lahlali R. Karunakaran C. Kumar S. Davis A. R. Bueckert R. A. (2015). Seed set, pollen morphology and pollen surface composition response to heat stress in field pea. Plant Cell Environ. 38, 2387–2397. doi: 10.1111/pce.12589 26081983

[B71] Jiang Y. Lindsay D. L. Davis A. R. Wang Z. MacLean D. E. Warkentin T. D. . (2020). Impact of heat stress on pod-based yield components in field pea (*Pisum sativum* l.). J. Agron. Crop Sci. 206, 76–89. doi: 10.1111/jac.12365

[B72] Kültz D. (2003). Evolution of the cellular stress proteome: from monophyletic origin to ubiquitous function. J. Exp. Biol. 206, 3119–3124. doi: 10.1242/jeb.00549 12909693

[B73] Karr E. J. Linck A. J. Swanson C. A. (1959). The effect of short periods of high temperature during day and night periods on pea yields. Am. J. Bot. 46, 91–93. doi: 10.2307/2439463

[B74] Kaushal N. Awasthi R. Gupta K. Gaur P. Siddique K. H. M. Nayyar H. (2013). Heat-stress-induced reproductive failures in chickpea (*Cicer arietinum*) are associated with impaired sucrose metabolism in leaves and anthers. Funct. Plant Biol. 40, 1334. doi: 10.1071/FP13082 32481199

[B75] Kaushal N. Bhandari K. Siddique K. H. M. Nayyar H. (2016). Food crops face rising temperatures: an overview of responses, adaptive mechanisms, and approaches to improve heat tolerance. Cogent Food Agric. 2. doi: 10.1080/23311932.2015.1134380

[B76] Khan M. A. Asaf S. Khan A. L. Jan R. Kang S.-M. Kim K.-M. . (2020). Thermotolerance effect of plant growth-promoting *Bacillus cereus* SA1 on soybean during heat stress. BMC Microbiol. 20, 175. doi: 10.1186/s12866-020-01822-7 32571217 PMC7310250

[B77] Kilasi N. L. Singh J. Vallejos C. E. Ye C. Jagadish S. V. K. Kusolwa P. . (2018). Heat stress tolerance in rice (*Oryza sativa* l.): identification of quantitative trait loci and candidate genes for seedling growth under heat stress. Front. Plant Sci. 9. doi: 10.3389/fpls.2018.01578 PMC6221968 30443261

[B78] Kotak S. Larkindale J. Lee U. von Koskull-Döring P. Vierling E. Scharf K. D. (2007). Complexity of the heat stress response in plants. Curr. Opin. Plant Biol. 10, 310–316. doi: 10.1016/j.pbi.2007.04.011 17482504

[B79] Krajewski P. Bocianowski J. Gawłowska M. Kaczmarek Z. Pniewski T. Święcicki W. . (2012). QTL for yield components and protein content: a multienvironment study of two pea (*Pisum sativum* l.) populations. Euphytica 183, 323–336. doi: 10.1007/s10681-011-0472-4

[B80] Kregel K. C. (2002). Invited review: Heat shock proteins: modifying factors in physiological stress responses and acquired thermotolerance. J. Appl. Physiol. 92, 2177–2186. doi: 10.1152/japplphysiol.01267.2001 11960972

[B81] Kreplak J. Madoui M.-A. Cápal P. Novák P. Labadie K. Aubert G. . (2019). A reference genome for pea provides insight into legume genome evolution. Nat. Genet. 51, 1411–1422. doi: 10.1038/s41588-019-0480-1 31477930

[B82] Kumari T. Deka S. C. (2021). Potential health benefits of garden pea seeds and pods: A review. Legume Sci. 3. doi: 10.1002/leg3.82

[B83] Kumar U. Joshi A. K. Kumari M. Paliwal R. Kumar S. Röder M. S. (2010). Identification of QTLs for stay green trait in wheat (*Triticum aestivum* l.) in the ‘Chirya 3’ × ‘Sonalika’ population. Euphytica 174, 437–445. doi: 10.1007/s10681-010-0155-6

[B84] Kumar J. Mir R. R. Shafi S. Sen Gupta D. Djalovic I. Miladinovic J. . (2021). Genomics associated interventions for heat stress tolerance in cool season adapted grain legumes. Int. J. Mol. Sci. 23, 399. doi: 10.3390/ijms23010399 35008831 PMC8745526

[B85] Kumar J. Sen Gupta D. Djalovic I. (2020). Breeding, genetics, and genomics for tolerance against terminal heat in lentil: Current status and future directions. Legume Sci. 2. doi: 10.1002/leg3.38

[B86] Kumar S. Thakur P. Kaushal N. Malik J. A. Gaur P. Nayyar H. (2013). Effect of varying high temperatures during reproductive growth on reproductive function, oxidative stress and seed yield in chickpea genotypes differing in heat sensitivity. Arch. Agron. Soil Sci. 59, 823–843. doi: 10.1080/03650340.2012.683424

[B87] Lambert R. G. Linck A. J. (1958). Effects of high temperature on yield of peas. Plant Physiol. 33, 347–350. doi: 10.1104/pp.33.5.347 16655145 PMC541099

[B88] Lamichaney A. Parihar A. K. Hazra K. K. Dixit G. P. Katiyar P. K. Singh D. . (2021). Untangling the influence of heat stress on crop phenology, seed set, seed weight, and germination in field pea (*Pisum sativum* l.). Front. Plant Sci. 12. doi: 10.3389/fpls.2021.635868 PMC8040956 33854520

[B89] Larmure A. Munier-Jolain N. G. (2019). High temperatures during the seed-filling period decrease seed nitrogen amount in pea (*Pisum sativum* l.): evidence for a sink limitation. Front. Plant Sci. 10. doi: 10.3389/fpls.2019.01608 PMC6934051 31921254

[B90] Li G. Liu R. Xu R. Varshney R. K. Ding H. Li M. . (2022b). Development of an agrobacterium-mediated CRISPR/Cas9 system in pea (*Pisum sativum* l.). Crop J. doi: 10.1016/j.cj.2022.04.011

[B91] Liu Y. Li J. Zhu Y. Jones A. Rose R. J. Song Y. (2019). Heat stress in legume seed setting: effects, causes, and future prospects. Front. Plant Sci. 10. doi: 10.3389/fpls.2019.00938 PMC6684746 31417579

[B92] Liu Y.-H. Offler C. E. Ruan Y.-L. (2013). Regulation of fruit and seed response to heat and drought by sugars as nutrients and signals. Front. Plant Sci. 4. doi: 10.3389/fpls.2013.00282 PMC3729977 23914195

[B93] Li Y. Wu X. Zhang Y. Zhang Q. (2022a). CRISPR/Cas genome editing improves abiotic and biotic stress tolerane in crops. Front. Genome Ed. 4. doi: 10.3389/fgeed.2022.987817 PMC9524261 36188128

[B94] Lopes-Caitar V. S. de Carvalho M. C. Darben L. M. Kuwahara M. K. Nepomuceno A. L. Dias W. P. . (2013). Genome-wide analysis of the hsp 20 gene family in soybean: comprehensive sequence, genomic organization and expression profile analysis under abiotic and biotic stresses. BMC Genomics 14, 577. doi: 10.1186/1471-2164-14-577 23985061 PMC3852298

[B95] Lucas M. R. Ehlers J. D. Huynh B.-L. Diop N.-N. Roberts P. A. Close T. J. (2013). Markers for breeding heat-tolerant cowpea. Mol. Breed. 31, 529–536. doi: 10.1007/s11032-012-9810-z

[B96] Lu L. Liu H. Wu Y. Yan G. (2022). Wheat genotypes tolerant to heat at seedling stage tend to be also tolerant at adult stage: The possibility of early selection for heat tolerance breeding. Crop J 1006–1013. doi: 10.1016/j.cj.2022.01.005

[B97] Mahoney J. (1991). “Field pea,” in In: New crops: agronomy and potential of alternative crop species (Melbourne, Australia: Inkata Press), 53–62.

[B98] Mammadov J. Buyyarapu R. Guttikonda S. K. Parliament K. Abdurakhmonov I. Y. Kumpatla S. P. (2018). Wild relatives of maize, rice, cotton, and soybean: treasure troves for tolerance to biotic and abiotic stresses. Front. Plant Sci. 9. doi: 10.3389/fpls.2018.00886 PMC6032925 30002665

[B99] Mansfield M. A. Key J. L. (1987). Synthesis of the low molecular weight heat shock proteins in plants. Plant Physiol. 84, 1007–1017. doi: 10.1104/pp.84.4.1007 16665553 PMC1056718

[B100] Marfo K. O. Hall A. E. (1992). Inheritance of heat tolerance during pod set in cowpea. Crop Sci. 32, 912–918. doi: 10.2135/cropsci1992.0011183X003200040015x

[B101] McDonald G. K. Paulsen G. M. (1997). High temperature effects on photosynthesis and water relations of grain legumes. Plant Soil. 196, 47–58. doi: 10.1023/A:1004249200050

[B102] Medina E. Kim S.-H. Yun M. Choi W.-G. (2021). Recapitulation of the function and role of ROS generated in response to heat stress in plants. Plants 10, 371. doi: 10.3390/plants10020371 33671904 PMC7918971

[B103] Mendel G. (1865). “Versucheüber plflanzenhybriden,” in Verhandlungen des naturforschenden vereines in brünn, bd. IV fur das jahr, abhandlungen, 3–47.

[B104] Mohapatra C. Chand R. Tiwari J. K. Singh A. K. (2020). Effect of heat stress during flowering and pod formation in pea (*Pisum sativum* l.). Physiol. Mol. Biol. Plants 26, 1119–1125. doi: 10.1007/s12298-020-00803-4 32549677 PMC7266882

[B105] Munier-Jolain N. Carrouée B. (2010). “Physiology of the pea crop,” (Boca, Raton: Science publisher, marketed and distributed by CRC Press). doi: 10.1201/b10504

[B106] Nemeskeri E. (2004). Heat tolerance in grain legumes. Bodenkultur 55, 3–11.

[B107] Nijabat A. Bolton A. Mahmood-ur-Rehman M. Shah A. I. Hussain R. Naveed N. H. . (2020). Cell membrane stability and relative cell injury in response to heat stress during early and late seedling stages of diverse carrot (*Daucus carota* l.) germplasm. HortScience 55, 1446–1452. doi: 10.21273/HORTSCI15058-20

[B108] Ni Z. Li H. Zhao Y. Peng H. Hu Z. Xin M. . (2018). Genetic improvement of heat tolerance in wheat: recent progress in understanding the underlying molecular mechanisms. Crop J. 6, 32–41. doi: 10.1016/j.cj.2017.09.005

[B109] Nonnecke I. L. Adedipe N. O. Ormrod D. P. (1971). Temperature and humidity effects on the growth and yield of pea cultivars. Can. J. Plant Sci. 51, 479–484. doi: 10.4141/cjps71-094

[B110] Ofir M. Gross Y. Bangerth F. Kigel J. (1993). High temperature effects on pod and seed production as related to hormone levels and abscission of reproductive structures in common bean (*Phaseolus vulgaris* l.). Sci. Hortic. (Amsterdam). 55, 201–211. doi: 10.1016/0304-4238(93)90032-L

[B111] Osorio E. E. Davis A. R. Bueckert R. (2021). High temperature disturbs ovule development in field pea (*Pisum sativum* l.). Botany 100, 47–61. doi: 10.1139/cjb-2021-0078

[B112] Ozga J. A. Kaur H. Savada R. P. Reinecke D. M. (2016). Hormonal regulation of reproductive growth under normal and heat-stress conditions in legume and other model crop species. J. Exp. Bot. 68 (8), 1885–1894, erw464. doi: 10.1093/jxb/erw464 28011717

[B113] Paul P. Samineni S. Thudi M. Sajja S. Rathore A. Das R. . (2018). Molecular mapping of QTLs for heat tolerance in chickpea. Int. J. Mol. Sci. 19, 2166. doi: 10.3390/ijms19082166 30044369 PMC6121679

[B114] Poggio S. L. Satorre E. H. Dethiou S. Gonzalo G. M. (2005). Pod and seed numbers as a function of photothermal quotient during the seed set period of field pea (*Pisum sativum*) crops. Eur. J. Agron. 22, 55–69. doi: 10.1016/j.eja.2003.12.003

[B115] Pottorff M. Roberts P. A. Close T. J. Lonardi S. Wanamaker S. Ehlers J. D. (2014). Identification of candidate genes and molecular markers for heat-induced brown discoloration of seed coats in cowpea [*Vigna unguiculata* (L.) walp]. BMC Genomics 15, 328. doi: 10.1186/1471-2164-15-328 24885083 PMC4035059

[B116] Pradhan G. P. Xue Q. Jessup K. E. Rudd J. C. Liu S. Devkota R. N. . (2014). Cooler canopy contributes to higher yield and drought tolerance in new wheat cultivars. Crop Sci. 54, 2275–2284. doi: 10.2135/cropsci2013.11.0788

[B117] Prasad P. V. V. Bheemanahalli R. Jagadish S. V. K. (2017). Field crops and the fear of heat stress–opportunities, challenges and future directions. F. Crop Res. 200, 114–121. doi: 10.1016/j.fcr.2016.09.024

[B118] Prasad P. V. V. Pisipati S. R. Mutava R. N. Tuinstra M. R. (2008). Sensitivity of grain sorghum to high temperature stress during reproductive development. Crop Sci. 48, 1911–1917. doi: 10.2135/cropsci2008.01.0036

[B119] Pratap A. Gupta S. Nair R. Gupta S. Schafleitner R. Basu P. . (2019). Using plant phenomics to exploit the gains of genomics. Agronomy 9, 126. doi: 10.3390/agronomy9030126

[B120] Priya M. Dhanker O. P. Siddique K. H. M. HanumanthaRao B. Nair R. M. Pandy S. . (2019). Drougth and heat stress-related proteins: an update about their functional relevane in imparting stress tolerance in agricultural crops.Theor. Appl. Gent. 132, 1607–1638. doi: 10.1007/s00122-019-03331-2. 30941464

[B121] Pumphrey F. V. Ramig R. E. (1990). Field response of peas to excess heat during the reproductive stage of growth. J. Am. Soc Hortic. Sci. 115, 898–900.

[B122] Ramakrishna G. Kaur P. Singh A. Yadav S. S. Sharma S. Singh N. K. . (2021). Comparative transcriptome analyses revealed different heat stress responses in pigeonpea (*Cajanus cajan*) and its crop wild relatives. Plant Cell Rep. 40, 881–898. doi: 10.1007/s00299-021-02686-5 33837822

[B123] Ridge P. E. Pye D. L. (1985). The effects of temperature and frost at flowering on the yield of peas grown in a Mediterranean environment. Field Crop Res. 12, 339–346. doi: 10.1016/0378-4290(85)90079-6

[B124] Ristic Z. Bukovnik U. Momcilovic I. Fu J. Prasad P. V. V. (2008). Heat-induced accumulation of chloroplast protein synthesis elongation factor, EF-tu, in winter wheat. J. Plant Physiol. 165, 192–202. doi: 10.1016/j.jplph.2007.03.003 17498838

[B125] Ruan Y. L. (2014). Sucrose metabolism: gateway to diverse carbon use and sugar signaling. Annu. Rev. Plant Biol. 65, 33–67. doi: 10.1146/annurev-arplant-050213-040251 24579990

[B126] Sadras V. O. Lake L. Leonforte A. McMurray L. S. Paull J. G. (2013). Screening field pea for adaptation to water and heat stress: Associations between yield, crop growth rate and seed abortion. F. Crop Res. 150, 63–73. doi: 10.1016/j.fcr.2013.05.023

[B127] Sarsu F. Ghanim A. M. A. Das P. Bahuguna R. N. Kusolwa P. M. Ashraf M. . (2018). Pre-field screening protocols for heat-tolerant mutants in rice (Cham: Springer International Publishing). doi: 10.1007/978-3-319-77338-4

[B128] Sehgal A. Sita K. Bhandari K. Kumar S. Kumar J. Prasad P. V. V. . (2019). Influence of drought and heat stress, applied independently or in combination during seed development, on qualitative and quantitative aspects of seeds of lentil (*Lens culinaris* medikus) genotypes, differing in drought sensitivity. Plant Cell Environ. 42, 198–211. doi: 10.1111/pce.13328 29744880

[B129] Sehgal A. Sita K. Siddique K. H. M. Kumar R. Bhogireddy S. Varshney R. K. . (2018). Drought or/and heat-stress effects on seed filling in food crops: impacts on functional biochemistry, seed yields, and nutritional quality. Front. Plant Sci. 9. doi: 10.3389/fpls.2018.01705 PMC6277783 30542357

[B130] Shah Z. Iqbal A. Khan F. U. Khan H. U. Durrani F. Ahmad M. Z. (2020). Genetic manipulation of pea (*Pisum sativum* l.) with *Arabidopsis*'s heat shock factor *HsfA1d* improves ROS scavenging system to confront thermal stress. Genet. Resour. Crop Evol. 67, 2119–2127. doi: 10.1007/s10722-020-00966-9

[B131] Shah N. H. Paulsen G. M. (2003). Interaction of drought and high temperature on photosynthesis and grain-filling of wheat. Plant Soil 257, 219–226. doi: 10.1023/A:1026237816578

[B132] Simões-Araújo J. L. Rodrigues R. L. de A. Gerhardt L. B. Mondego J. M. Alves-Ferreira M. Rumjanek N. G. . (2002). Identification of differentially expressed genes by cDNA-AFLP technique during heat stress in cowpea nodules. FEBS Lett. 515, 44–50. doi: 10.1016/S0014-5793(02)02416-X 11943192

[B133] Singh A. K. Srivastava C. P. (2015). Effect of plant types on grain yield and lodging resistance in pea. Indian J. Genet. Plant Breed. 75, 69–74. doi: 10.5958/0975-6906.2015.00008.5

[B134] Sita K. Sehgal A. Bhandari K. Kumar J. Kumar S. Singh S. . (2018). Impact of heat stress during seed filling on seed quality and seed yield in lentil (*Lens culinaris* medikus) genotypes. J. Sci. Food Agric. 98, 5134–5141. doi: 10.1002/jsfa.9054 29635707

[B135] Sita K. Sehgal A. HanumanthaRao B. Nair R. M. Prasad P. V. V. Kumar S. . (2017). Food legumes and rising temperatures: effects, adaptive functional mechanisms specific to reproductive growth stage and strategies to improve heat tolerance. Front. Plant Sci. 8. doi: 10.3389/fpls.2017.01658 PMC5662899 29123532

[B136] Srikanthbabu V. Ganeshkumar Krishnaprasad B. T. Gopalakrishna R. Savitha M. Udayakumar M. (2002). Identification of pea genotypes with enhanced thermotolerance using temperature induction response technique (TIR). J. Plant Physiol. 159, 535–545. doi: 10.1078/0176-1617-00650

[B137] Srivastava S. Yadav A. Seem K. Mishra S. Chaudhary V. Nautiyal C. S. (2008). Effect of high temperature on *Pseudomonas putida* NBRI0987 biofilm formation and expression of stress sigma factor RpoS. Curr. Microbiol. 56, 453–457. doi: 10.1007/s00284-008-9105-0 18219523

[B138] Stanfield B. Ormord D. P. Fletcher H. F. (1966). Response of peas to environment II, effects of temperature in controlled environment cabinets. Can. J. Plant Sci. 46, 195–203. doi: 10.4141/cjps66-029

[B139] Stevens M. A. Rudich J. (1978). Genetic potential for overcoming physiological limitations on adaptability, yield and quality in tomato. Hort Sci. 13, 673–678. doi: 10.21273/HORTSCI.13.6.673

[B140] Susmita C. Aghora T.S. M. N. B. R. M. (2020). Breeding for improvement of high temperature tolerance in garden pea (*Pisum sativum* l.) for off season cultivation. J. Hortl. Sci. 15, 62–66. doi: 10.24154/JHS.2020.v15i01.008

[B141] Tafesse E. G. (2018). Heat stress resistance in pea (Pisum sativum l.) based on canopy and leaf traits (Saskatchewan: University of Saskatchewan Saskatoon) 2018.

[B142] Tafesse E. G. Gali K. K. Lachagari V. B. R. Bueckert R. Warkentin T. D. (2020). Genome-wide association mapping for heat stress responsive traits in field pea. Int. J. Mol. Sci. 21, 2043. doi: 10.3390/ijms21062043 32192061 PMC7139655

[B143] Tafesse E. G. Gali K. K. Lachagari V. B. R. Bueckert R. Warkentin T. D. (2021). Genome-wide association mapping for heat and drought adaptive traits in pea. Genes (Basel). 12, 1897. doi: 10.3390/genes12121897 34946846 PMC8701326

[B144] Tafesse E. G. Warkentin T. D. Bueckert R. A. (2019). Canopy architecture and leaf type as traits of heat resistance in pea. F. Crop Res. 241, 107561. doi: 10.1016/j.fcr.2019.107561

[B145] Talalaiev O. Korduym E. (2014). Expression of small heat shock protein (sHSP) genes in the garden pea (*Pisum sativum*) under slow horizontal clinorotation. Plant Signal. Behav. 9, e29035. doi: 10.4161/psb.29035 24786104 PMC4091545

[B146] Tar’an B. Warkentin T. Somers D. J. Miranda D. Vandenberg A. Blade S. . (2003). Quantitative trait loci for lodging resistance, plant height and partial resistance to mycosphaerella blight in field pea (*Pisum sativum* l.). Theor. Appl. Genet. 107, 1482–1491. doi: 10.1007/s00122-003-1379-9 12920512

[B147] Tar’an B. Warkentin T. Somers D. J. Miranda D. Vandenberg A. Blade S. . (2004). Identification of quantitative trait loci for grain yield, seed protein concentration and maturity in field pea (*Pisum sativum* l.). Euphytica 136, 297–306. doi: 10.1023/B:EUPH.0000032721.03075.a0

[B148] Tayeh N. Aubert G. Pilet-Nayel M.-L. Lejeune-Hénaut I. Warkentin T. D. Burstin J. (2015). Genomic tools in pea breeding programs: status and perspectives. Front. Plant Sci. 6. doi: 10.3389/fpls.2015.01037 PMC4661580 26640470

[B149] Timmerman-Vaughan G. M. Mills A. Whitfield C. Frew T. Butler R. Murray S. . (2005). Linkage mapping of QTL for seed yield, yield components, and developmental traits in pea. Crop Sci. 45, 1336–1344. doi: 10.2135/cropsci2004.0436

[B150] Todorova D. Katerova Z. Shopova E. Jodinskiene M. Jurkoniene S. Sergiev I. (2016). Responses of pea plants to heat stress and spermine treatment. Zemdirbyste-Agriculture 103, 99–106. doi: 10.13080/z-a.2016.103.013

[B151] Verma D. Aghora T. S. Hunashikatti L. Sadashiva A. T. (2019). Screening of garden pea genotypes for high temperature tolerance using temperature induction response technique. Int. J. Curr. Microbiol. Appl. Sci. 8, 2065–2073. doi: 10.20546/ijcmas.2019.808.241

[B152] Vijaylaxmi (2013). Effect of high temperature on growth,biomass and yield of field pea. Leg. Res. 36, 250–254.

[B153] Wahid A. Gelani S. Ashraf M. Foolad M. (2007). Heat tolerance in plants: an overview. Environ. Exp. Bot. 61, 199–223. doi: 10.1016/j.envexpbot.2007.05.011

[B154] Wang D. Yang T. Liu R. Li N. Ahmad N. Li G. . (2022). Large-Scale heat-tolerance screening and genetic diversity of pea (*Pisum sativum* l.) germplasms. Plants 11 (19), 2473. doi: 10.3390/plants11192473 36235339 PMC9573610

[B155] Waraich E. Ahmad R. Halim A. Aziz T. (2012). Alleviation of temperature stress by nutrient management in crop plants: a review. J. Soil Sci. Plant Nutr. 12, 221–244. doi: 10.4067/S0718-95162012000200003

[B156] Weeden N. F. (2007). Genetic changes accompanying the domestication of pisum sativum: is there a common genetic basis to the “domestication syndrome“ for legumes? Ann. Bot. 100, 1017–1025. doi: 10.1093/aob/mcm122 17660515 PMC2759201

[B157] Will T. Schmidtberg H. Skaljac M. Vilcinskas A. (2017). Heat shock protein 83 plays pleiotropic roles in embryogenesis, longevity, and fecundity of the pea aphid. Acyrthosiphon pisum. Dev. Genes Evol. 227, 1–9. doi: 10.1007/s00427-016-0564-1 27743033 PMC5203865

[B158] Wood C. K. Pratt J. R. Moore A. L. (1998). Identification and characterisation of cultivar-specific 22-kDa heat shock proteins from mitochondria of pisum sativum. Physiol. Plant 103, 369–376. doi: 10.1034/j.1399-3054.1998.1030310.x

[B159] Xiao G. Zhang Q. Wang R. Yao Y. Zhao H. Bai H. . (2009). Effects of temperature increase on pea production in a semiarid region of China. Air Soil Water Res. 2, ASWR.S2488. doi: 10.4137/ASWR.S2488

[B160] Xue G.-P. Sadat S. Drenth J. McIntyre C. L. (2014). The heat shock factor family from *Triticum aestivum* in response to heat and other major abiotic stresses and their role in regulation of heat shock protein genes. J. Exp. Bot. 65, 539–557. doi: 10.1093/jxb/ert399 24323502 PMC3904712

[B161] Xu W. Rosenow D. T. Nguyen H. T. (2000). Stay green trait in grain sorghum: relationship between visual rating and leaf chlorophyll concentration. Plant Breed. 119, 365–367. doi: 10.1046/j.1439-0523.2000.00506.x

[B162] Yadav M. R. Choudhary M. Singh J. Lal M. K. Jha P. K. Udawat P. . (2022). Impacts, tolerance, adaptation, and mitigation of heat stress on wheat under changing climates. Int. J. Mol. Sci. 23, 2838. doi: 10.3390/ijms23052838 35269980 PMC8911405

[B163] Zhang Y. Lou H. Guo D. Zhang R. Su M. Hou Z. . (2018). Identifying changes in the wheat kernel proteome under heat stress using iTRAQ. Crop J. 6, 600–610. doi: 10.1016/j.cj.2018.04.003

[B164] Zhang X. Rerksiri W. Liu A. Zhou X. Xiong H. Xiang J. . (2013). Transcriptome profile reveals heat response mechanism at molecular and metabolic levels in rice flag leaf. Gene 530, 185–192. doi: 10.1016/j.gene.2013.08.048 23994682

[B165] Zhang C. Tar’an B. Warkentin’ T. Tullu A. Bett K. E. Vandenberg B. . (2006). Selection for lodging resistance in early generations of field pea by molecular markers. Crop Sci. 46, 321–329. doi: 10.2135/cropsci2005.0123

[B166] Zhao C. Liu B. Piao S. Wang X. Lobell D. B. Huang Y. . (2017). Temperature increase reduces global yields of major crops in four independent estimates. Proc. Natl. Acad. Sci. 114, 9326–9331. doi: 10.1073/pnas.1701762114 28811375 PMC5584412

[B167] Zhu B. Ye C. Lü H. Chen X. Chai G. Chen J. . (2006). Identification and characterization of a novel heat shock transcription factor gene, *GmHsfA1*, in soybeans (*Glycine max*). J. Plant Res. 119, 247–256. doi: 10.1007/s10265-006-0267-1 16570125

